# A Physicochemical Consideration of Prebiotic Microenvironments for Self-Assembly and Prebiotic Chemistry

**DOI:** 10.3390/life12101595

**Published:** 2022-10-13

**Authors:** Arpita Saha, Ruiqin Yi, Albert C. Fahrenbach, Anna Wang, Tony Z. Jia

**Affiliations:** 1 Blue Marble Space Institute of Science, 600 1st Ave, Floor 1, Seattle, WA 98104, USA; 2 Amity Institute of Applied Sciences, Amity University, Kolkata 700135, India; 3 Earth-Life Science Institute, Tokyo Institute of Technology, 2-12-1-IE-1 Ookayama, Meguro-ku, Tokyo 152-8550, Japan; 4 School of Chemistry, UNSW Sydney, Sydney, NSW 2052, Australia; 5 Australian Centre for Astrobiology, UNSW Sydney, Sydney, NSW 2052, Australia; 6 UNSW RNA Institute, UNSW Sydney, Sydney, NSW 2052, Australia

**Keywords:** origin of life, chemical evolution, reaction microenvironments, physical chemistry, geochemistry

## Abstract

The origin of life on Earth required myriads of chemical and physical processes. These include the formation of the planet and its geological structures, the formation of the first primitive chemicals, reaction, and assembly of these primitive chemicals to form more complex or functional products and assemblies, and finally the formation of the first cells (or protocells) on early Earth, which eventually evolved into modern cells. Each of these processes presumably occurred within specific prebiotic reaction environments, which could have been diverse in physical and chemical properties. While there are resources that describe prebiotically plausible environments or nutrient availability, here, we attempt to aggregate the literature for the various physicochemical properties of different prebiotic reaction microenvironments on early Earth. We introduce a handful of properties that can be quantified through physical or chemical techniques. The values for these physicochemical properties, if they are known, are then presented for each reaction environment, giving the reader a sense of the environmental variability of such properties. Such a resource may be useful for prebiotic chemists to understand the range of conditions in each reaction environment, or to select the medium most applicable for their targeted reaction of interest for exploratory studies.

## 1. Introduction

The early Earth was like a laboratory but without an intervening chemist. That is to say, early Earth possessed a variety of chemicals, reaction vessels/compartments, and conditions [1], generating complex chemical systems without a target, but which managed to self-organize into life. For many of these scenarios, interdisciplinary studies have been carried out to qualify and quantify their prebiotic plausibility. For example, the origins of life community have considered different geological settings (i.e., hot springs or oceans) as plausible “reaction vessels” on early Earth [2,3,4,5]. However, these geological settings, while informative about constraining the potential reaction environments, conditions, and chemicals, are mostly still at large length scales of centimeters, meters, or kilometers. Less attention has been paid to early Earth environments at the microscopic level, the length scales that have the potential to directly affect the dynamics of chemical reactions, self-assembly, and cellular/protocellular processes.

Indeed, within larger geological settings, a variety of physical and chemical environments exist at smaller length scales. These smaller ‘microenvironments’, which are as small as the microliter or micron scale, could vary significantly in physical and chemical properties. Examples include the widely varying temperature and pH conditions in hot springs [6] or the simultaneous existence of both aqueous environments as well as supercritical liquid carbon dioxide (CO_2_) in the deep ocean [7].

**Figure 1:**
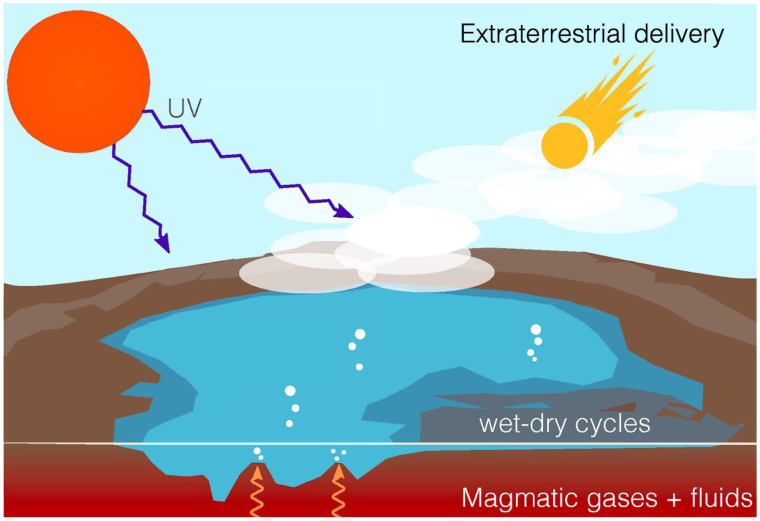
A variety of chemical processes could have occurred on early Earth. Prebiotic reactions could have occurred in the gas phase (atmospheric synthesis), the aqueous phase, or on material interfaces, just to name a few. Other reactions could have occurred extraterrestrially, followed by delivery to Earth; in this review, we particularly focus on condensed phase reactions on Earth. Figure adapted and reprinted with permission from [9] under a Creative Commons license.

Here, we consider a number of prebiotic microenvironments on Earth and discuss their physical and chemical characteristics and subsequent impact on primitive reactions and/or self-assembly. The intention is to create a resource that researchers can use to guide their laboratory reactions toward more realistic geological conditions or to use specific reaction microenvironments to constrain what prebiotic processes can take place. This review focuses on condensed phase reactions including but not limited to aqueous phase chemistry, interfacial chemistry, and hydrothermal/geochemical synthesis (Figure 1) [1]. We refer the reader to reviews of gas-phase chemistries and in particular, gas-phase photochemistry [8,9,10].

The scope of this paper is primarily on environments generated purely from and/or residing in geological formations, although a number of potential environments generated from self-assembled primitive molecules (such as in the interior of vesicle bilayers [11] or within phase-separated polymer droplets [12,13]) are briefly mentioned. Although a number of reaction environments exist extraterrestrially such as methane surface lakes or ammonium-rich sub-surface lakes on Titan [14], we restrict the environments covered here to only those thought to have occurred on Earth. In the future, we hope to expand this analysis to more extraterrestrial environments as more concrete information on the chemical and physical properties of such environments becomes known, following additional planetary science studies and missions such as the Titan Dragonfly mission [15]. We refer the interested reader to papers discussing prebiotic chemistry in extraterrestrial environments such as meteorites [16], interstellar ice [17] or gas [18], or other planetary bodies [2,19,20,21].

## 2. Prebiotic Microenvironments and Where to Find Them

The goal in this section is to take a physicochemical perspective and categorize specific reaction microenvironments available on early Earth, which may be found within a number of (macroscopic) geological environments and scenarios. The overview of the physicochemical properties is expected to help point to opportunities that different environments present. For completeness, we encourage the reader to consider how the geological context is inextricable from prebiotic chemistry by reading the excellent works on the topic (e.g., [1,22,23,24,25]).

We start our overview with bulk polar and non-polar environments. Within these broad categories, different colloidal structures are discussed. Finally, we end with a brief discussion of molten and solid rocks.

### 2.1. Aqueous Environments

Living processes depend on water. One reason is that liquid water is important for diffusion, thus allowing reactants to meet. Water also hosts acid and base chemistries, and is a polar protic solvent that can readily solubilize ionic compounds and hydrogen bond with solutes. Bulk water’s highly polar environment is responsible for the hydrophobic effect [26], liquid–liquid phase separation [27], and promotes amphiphile self-assembly into micelles, membranes, or other structures [28].

On early Earth, just as in the present day, there would have been sources of fresh water including streams, ponds, lakes, hot springs as well as salt water such as oceans and hydrothermal systems containing brines. The range of different salinities is covered in Section 3.1.

#### 2.1.1. Bulk Aqueous Solution

In bulk systems there are still microenvironments that should be taken into account. One important factor to consider is the temporal stability of the aqueous environment. Surficial systems can be subjected to wet/dry or freeze/thaw cycles, and while this is not applicable to larger bodies of water, turbulent mixing still creates temporal instability in any aqueous system where the length scales are larger than the Reynolds number. That said, ocean stratification can broadly lead to different zones of stable salt/density and temperature, with other variables such as nutrient content potentially varying during mixing [29]. If the relevant length scales are small and the velocities are slow such as in rock pores or narrow channels [30], the flow could easily be laminar, and other types of transport processes such as thermophoresis [30,31] or diffusiophoresis [32] could begin to dominate.

#### 2.1.2. Sea Spray (Aqueous Aerosols)

Another form of liquid aqueous environments is in aerosol droplets [33], which could have formed on early Earth due to turbulent waves or wind acting on different bodies of water. While the volumes of water in aerosols are small, the high surface area to volume ratio and ample exposure to sunlight means that surface-based processes and photochemistry can generate new molecules potentially not synthesizable in other environments [33,34] affording, for example, cross-linked lipids [35].

#### 2.1.3. Gels and Other Hygroscopic Environments

Aqueous environments can also exist in forms that are intermediates to the solid and liquid phases. Siliceous hot-spring deposits, which likely would have been present on early Earth, contain hydrophilic amorphous silica that can retain water [36]. Mixtures of organic molecules likely present on early Earth have also been shown to form gels [37,38]. Hygroscopic salts also sequester water [39]. These environments enable unique aqueous-phase chemistries such as the synthesis of polymer-supported zinc sulfide nanocrystals [40] or photochemical phosphorylation [38], and can also reduce evaporation rates and prevent total desiccation.

#### 2.1.4. Ice

The final form of an aqueous-derived environment considered here is ice, which could have been derived from the freezing of liquid aqueous solutions in different bodies of water on early Earth. While carbon-cycle modeling reveals that early Earth is thought to have been temperate [4], fluctuations in conditions could have potentially created sub-zero temperatures and thus ice. Eutectic phases in ice provide aqueous environments that are concentrated in solutes, leading to reactions that are unfavorable in a dilute aqueous environment; we cover eutectic phases in Section 2.2.1.

### 2.2. Alternative Liquid Environments

#### 2.2.1. Non-Aqueous Solvents

Aside from water, other liquid environments on early Earth could have been in the form of non-aqueous solvents. Many reactions in prebiotic chemistry are formally known as condensations, which are reactions that covalently join together two compounds while eliminating a molecule of water in the process. The polymerization of amino acids and ribonucleotides into peptides and RNA, respectively, are examples of condensation reactions. In water, these condensations tend not to be spontaneous, partly as a consequence of Le Chatelier’s principle, since water as a solvent is present in large excess and pushes the equilibrium towards the reactants. Hence, non-aqueous solvents (i.e., those based on organic compounds) have the potential to make condensation reactions more favorable.

While the modern synthetic organic laboratory has a large variety of non-aqueous (organic) solvents at its disposal, the majority would almost certainly not have been abundantly available on early Earth. One reason is simply the lack of prebiotically plausible synthetic pathways to achieve reservoir amounts of these organic compounds necessary to act as solvents, many of the chemical structures of which can be relatively complex. Another reason is that the temperature and pressure conditions of early Earth limit what potential solvents could have accumulated, even if endogenous prebiotic synthetic pathways were producing them in large quantities. The boiling points could be too low to exist as liquids at room temperature or be significantly lower than that of water so that their concentration from aqueous solutions is not realistic. Only organic liquids that have a higher boiling point than water could have accumulated to excess amounts, a circumstance required for a compound to act as a solvent. With these constraints in mind, it is conceivable, however, that some organic liquids could have accumulated in relatively large excess.

For example, an organic solvent that may have accumulated in certain early Earth geological scenarios is formamide. A formal hydration product of hydrogen cyanide (molecular formula: HCONH_2_), formamide has a boiling point of 210 °C under standard pressure and has limited azeotropic associations with water [41]. Formamide could have been produced through multiple pathways [42] including mechanisms that involve atmospheric spark-discharge [43], ionizing radiation such as proton irradiation [44], UV irradiation [45], pyrolysis [46], or thermal reactions promoted by catalysis [47]. While it is unclear whether very large pools of formamide could have existed on early Earth (at least in comparison to aqueous pools), even transient accumulation of small volumes of formamide in different microenvironments on early Earth such as those that could occur in rock pores or on mineral surfaces following radiolytic synthesis and dehydration could have produced segregated organic formamide microenvironments that housed chemical reactions at the microscale [42].

#### 2.2.2. Deep Eutectic Solvents

Deep eutectic solvents (DESs) have also been recently considered as alternative non-aqueous liquids in prebiotic chemistry and could have formed readily, for example, within different ice–water systems on early Earth [48]. A eutectic solvent is a liquid made from a specific mixture of two or more substances that taken individually exist as solids, but as a mixture forms a liquid that has a single melting point lower than either of its individual components. The eutectic is the specific ratio of component compounds that exhibits the lowest melting point. A *deep* eutectic solvent is a mixture of solids whose melting point becomes so depressed that it exists as a liquid at room temperature. For example, a 1:2 ratio of choline chloride (melting point = 302 °C) to urea (melting point = 133 °C) has a melting point of 12 °C, and thus is a liquid at room temperature [49]. The mechanism of melting point depression is thought to involve hydrogen bonds, and so the majority of known DES mixtures involve hydrogen bond donors and acceptors [50]. DESs have the characteristics of high viscosity, low volatility, and are typically polar enough to dissolve high concentrations of ionic compounds. These non-aqueous solvents have been shown to promote various prebiotic condensation reactions including phosphorylation [51] and peptide bond formation [51,52]. Some of the components of typical DES mixtures [53,54] such as urea [55], glycerol [56], or acetamide [57] are also organic molecules thought to be generally available in prebiotic chemical systems.

#### 2.2.3. High Pressure Supercritical Fluids (CO_2_, H_2_O)

One alternative non-polar environment is supercritical fluids, found in the high pressure environments of deep ocean ridges and hydrothermal systems that likely also existed on early Earth [7]. Whilst supercritical CO_2_ has traditionally been thought of as rather non-polar by some in the scientific community, this, in fact, is incorrect; the polarity of supercritical CO_2_ can be tuned, and CO_2_ in this phase can also act either as a Lewis acid or a Lewis base depending on the specific conditions [58]. Conversely, water when supercritical becomes as non-polar as 1-dodecanol [59].

#### 2.2.4. Tars

Non-polar environments can also be found in tars, which can be made as a product of polymerization reactions containing prebiotically available organics, and result in thick, sticky substances that exhibit extremely slow diffusion times that, even in the presence of rainfall or aqueous solutions, is practically impossible to dilute [60,61]. From a microscopic perspective, it is a reaction environment that is ‘hard to leave’, but offers high concentrations and extremely complex reaction environments. Some molecules, however, are able to exit through the surface by sublimation or slowly leach out into surrounding fluids.

#### 2.2.5. Inside Lipid Bilayers and Related Interfacial Assemblies

The polar nature of water can induce amphiphilic molecules to self-assemble into a variety of phases including micelles, cubic phases, lamellar phases, and liposome or lipid bilayer vesicles. Vesicles and micelles, in particular, have been proposed to be primitive compartments that were precursors to modern cells (i.e., protocells) that could have assembled on early Earth, and the presence of such amphiphilic molecular assemblies on early Earth means that an aqueous environment can host non-polar compounds and thus non-aqueous chemistries. For a review of how these environments could affect different reactions, please see [62].

Amphiphilic molecules can also reduce surface energies [63] by adsorbing onto surfaces such as that of mineral particles [64] or exist at liquid–gas interfaces [35]. The importance of creating such layers is apparent in chemistry. Lipid monolayers have been shown to nucleate mineral growth [65], and surfaces can assist in creating desirable lipid membrane structures [66,67]. The intermembrane spaces could also be a potential site for a range of reactions, including RNA polymerization [68], where confinement to two dimensions and a non-aqueous environment is beneficial.

#### 2.2.6. Condensed Droplet Microenvironments

Non-amphiphilic molecules can also self-aggregate via non-covalent interactions into condensed phases. These condensed phases could form due to a process known as liquid–liquid phase separation, a common phenomenon in cells that forms membraneless organelles [13]. Such phase separation could have also occurred on early Earth and would have yielded membraneless droplets that can form associatively such as coacervation between nucleic acids and cationic peptides [12,69,70,71], or dissociatively such as aqueous two-phase systems [72,73,74] or polyester microdroplets [75,76,77]. These droplets can host and thus concentrate molecules via similar forces as the forces that lead to condensation [78,79]. The interior of such droplet microenvironments can also vary from apolar (mainly) polymer-based environments such as in polyester microdroplets [75] to polar aqueous (but polymer-rich) environments such as in coacervates [70].

### 2.3. Minerals/Rocks

#### 2.3.1. Solid Mineral Surfaces

Solid minerals are found all over the Earth’s crust in rock or suspended colloidal forms, and would have been present in abundance very early on in Earth’s history. They are capable of increasing the local concentration of molecules via adsorption due to electrostatics or by reducing interfacial energies [80,81,82,83]. Mineral surfaces can also preorganize molecules while precluding water to increase reaction rates [84]. As a physical environment, minerals can contain large surface area to volume ratios, with much of the area being internal 2D interlayers such as in clays, or narrow networks of rock pores that are shielded from light as well as turbulent flow. For an overview of the importance of minerals for prebiotic chemistry, we refer readers to [85,86,87,88,89,90].

Minerals are also an important source of elements essential to prebiotic chemistry such as phosphorous [91,92]. Consequently, minerals create local microenvironments that can not only enrich molecules by adsorption, but also leach out materials to their surroundings. In particular, we note that most chemical reactions involving minerals will occur at the mineral surface, i.e., a mineral-air or mineral-water interface such as within mineral pores or cracks [87,93,94]. However, there are some mineral-based chemical processes that could occur exclusively in the solid phase, such as metamorphic changes in rocks at high temperature and pressure, which could affect the availability of certain minerals.

#### 2.3.2. Mantle

Earth’s solid mantle, which would also have formed very early on in Earth’s history, is a source of minerals and gases that can partake in other chemistries once ejected/erupted onto the Earth’s surface [95], and undergoes solid-state convection, a key to plate tectonics [96]. The oxidation state of the mantle is possibly driven by the disproportionation of Fe^2+^ [97], with metallic iron sinking and Fe^3+^ persisting in the mantle, rendering it oxidizing [98]. It is the high viscosity and physical inaccessibility of the mantle that enables it to be transiently out of equilibrium with the ocean, atmosphere, and crust. As a result, the oxidation state, not to mention the temperatures and pressures, can differ vastly from other regions of Earth, and enable novel (inorganic) chemistries within the mantle [99,100].

## 3. Physicochemical Properties

In this section, we broadly introduce the relevant physicochemical characteristics that have wide variability amongst the reaction environments introduced above. The aggregated data showing the values of each physicochemical characteristic serve to guide the design of future prebiotic chemistry studies as a way for researchers to better understand the relevance of each reaction environment to different chemical processes.

### 3.1. Ionic Strength

Ionic strength is, simplistically, the total concentration of charge (both positive and negative) contributed by all dissolved ions in a given solution [101]. Ionic strength contributions are proportional to the square of the charge on the ion, and are thus greater for divalent ions compared to monovalent ions.

Ionic strength affects the solubility of electrolytes, inter- and intramolecular supramolecular interactions, the dissociation constant of acids (which can result in more dissolved protons in solution and lower pH [102]), and the strength of electrostatic interactions [103]. It can also impact the osmotic pressure of semipermeable systems. It may be significant that no living cell today has an intracellular concentration of 0.6 M NaCl, the sodium chloride concentration of the ocean. Instead, most cells use active transport to maintain the internal concentration of NaCl at 0.015 M, while KCl is maintained at approximately 0.15 M within the cell [104].

High ionic strengths could result in the dissociation of molecular complexes bound through charge-charge interactions such as peptide-nucleotide complexes that form primitive phase separated coacervates upon binding [69]. Salt can also inhibit the self-assembly of phospholipids into vesicles.

Here, we report the range of ionic strengths found in each of the prebiotic reaction environments introduced above (Table 1).

### 3.2. Surface Effects

**Figure 2:**
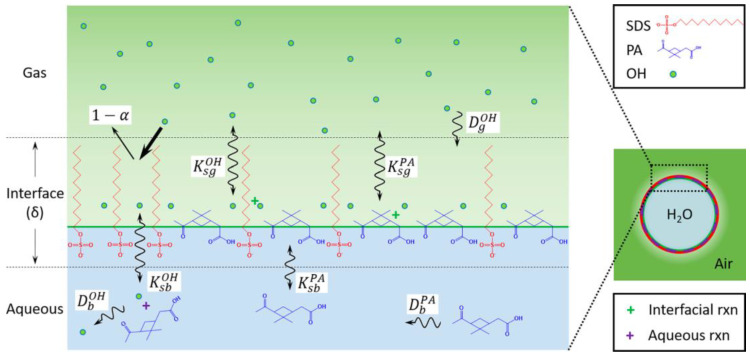
In a droplet system (blue) containing pinonic acid (PA), sodium dodecyl sulfate (SDS), and hydroxyl radicals (OH) in both aqueous and gas phases (green), various reactions can occur in the droplet (volume-dominated process), the gas phase (volume-dominated process), or the gas-droplet interface (surface-dominated process). For example, SDS participates only in reactions with OH (oxidation) at surface-dominated processes due to its high surface activity (as an amphiphile). However, PA can react with OH (oxidation) both at the air-droplet interface (surface-dominated process) as well as inside the bulk droplet (volume-dominated process) due to its lower surface activity than SDS. OH can also participate in reactions in the gas phase, the liquid phase, or at the interface. Reprinted with permission from Huang, Y. et al. “Probing the OH Oxidation of Pinonic Acid at the Air-Water Interface Using Field-Induced Droplet Ionization Mass Spectrometry (FIDI-MS)”. *J. Phys. Chem A*. **122**(31), 6445–6456 (2018). [115] Copyright 2018 American Chemical Society.

When considering microenvironments, surface effects must be taken into account because of the large surface area to volume ratios of such environments compared to the bulk. Whether that interface is solid-gas, liquid-gas, liquid-solid, or liquid-liquid, the interface could be a non-negligible site that concentrates materials and increases chemical reactivity [63]. This concentration mechanism could be relevant to prebiotic chemical reactions where the reactants are highly diluted in a mixed reactant pool and would otherwise not react to any appreciable degree [114]. The reactions that occur at the air–water interface of an aerosol or droplet could therefore be more important than reactions in the bulk of the aerosol/droplet [33,115] (Figure 2).

There are several mechanisms by which materials can accumulate at liquid interfaces. A hydrodynamic mechanism is the ‘coffee ring effect’. For example, gas bubbles within heated rock pores have been shown to concentrate catalytic nucleic acids at the bubble interface and increase catalytic activity [116]. This effect can also be driven by surface tensions. Because any surface or interface has a non-zero interfacial tension at the boundary (e.g., the air-water interface being ~72.8 mN/m at room temperature), materials have a propensity to adsorb to the boundary layer, which results in an overall lower free energy. Take, for example, amphiphilic molecules, which form monolayers at the liquid-liquid interfaces that can decrease the interfacial energy by an order of magnitude [63]. Finally, surfaces can adsorb molecules directly. Those studying chemical reactions in/around rock pores need to consider the effects of the mineral surface (e.g., roughness, chemical properties) on each species participating in the reaction [93,117].

Surface features and effects are specific to each system and are not explicitly presented here. Large surface areas are also implicit in microenvironments. For more information, we refer the interested reader to the interfacial catalysis literature [118,119,120].

### 3.3. Viscosity

Chemical reactions in solution are either diffusion-limited (where reactants will react instantaneously upon contact with each other, and the reaction is thus controlled by the speed at which the reactants diffuse toward each other in solution) or reaction-limited (e.g., due to some energetic barrier) [121].

For diffusion-limited reactions, the viscosity of materials will control the speed of the reaction with higher viscosities typically slowing down reaction rates. In some cases, high viscosity may aid reactions by limiting how far molecules can diffuse from each other. Highly viscous media have been shown to support the replication and catalysis of primitive nucleic acids [48,122].

Here, we report the typical viscosities found in each of the prebiotic environments introduced above (Table 2). It should be noted that the rheology of materials (how materials deform and flow) depends on the applied stresses and strains, the temperature, and the length scales considered. Gels, for instance, can appear solid at larger length scales but still support flow inside their pores. Some materials exhibit viscous propreties at long-time scales, and elastic behavior at short-time scales.

### 3.4. Specific Heat Capacity

Specific heat capacity is the amount of energy needed to increase the temperature of one kg of a material by one degree K. In other words, it can be used as a measure of the energy that it takes for a volume of a material to heat or cool down to a given temperature or the insulation or conduction ability of the material [139], and depends on a material’s temperature and phase.

The specific heat capacity is important where temperature stability (or fluctuations) is critical. Given that the heat capacity of water (a good insulator) [140] and solids such as rocks (which could be good heat conductors) [141] are quite different, their close proximity in the form of water-rock interfaces (such as in hydrothermal vent environments or hot springs [142]) leads to significant heat transfer and could potentially affect processes such as self-assembly [87], geoelectrochemistry [143], transport in thermal gradients [30], evaporation, or even mineral composition [144].

Here, we report on the range of specific heat found in each of the prebiotic environments introduced above (Table 3).

### 3.5. pH

Because pH is defined as a solution property, only liquids can exhibit a pH. pH is usually defined in terms of the autoionization of water, but the concept of pH can also be extended to neat nonaqueous solvents as long as they have some ability to donate a proton [160]. The solution pH affects chemical properties such as the protonation state of molecules in the solution and hence their potential to participate in chemical reactions or assembly into supramolecular structures. For example, pH fluctuations could give rise to the cyclical assembly and disassembly of coacervate droplets due to changes in the charge states of the constituent polymers [161], while pH changes also modulate RNA base-pairing, resulting in the ability to affect strand separation [162] and vesicle self-assembly [163]. While the pH of a solution is generally uniform, there are some cases where the pH within an environment exhibits changes and is not uniform such as in certain terrestrial lakes [164] or water mixing zones [165].

Here, we report on the range of pH found in each of the prebiotic environments introduced above (Table 4).

### 3.6. Density

The density of reaction environments is important to consider, because differences in density could lead to the physical separation of different components, a process that occurs during ocean stratification or hydrodynamic sorting.

While solids are generally more dense than liquids, which are more dense than gases, there are cases where this is inverted such as tungsten hexafluoride gas [182] being at least 10 times denser than graphene aerogel solid [183] or solid pumice being able to float on water. The density of a material will increase upon increasing pressure (decreasing the volume due to pressure-driven compression (Section 3.10)), while increasing temperatures will usually, but not always [184], cause a density decrease. Some materials are non-uniform (e.g., rocks or minerals [185]), leading to different microenvironments even within the same material.

Here, we report on the density of each of the prebiotic environments introduced above (Table 5); however, as there are different environments on early Earth with variable temperatures (such as hot springs [6]) and pressured (such as near hydrothermal vent systems in the deep ocean [186]), the densities reported here may change accordingly.

### 3.7. Dielectric Constant

A general adage in chemistry is that “like dissolves like”; polar solvents are more likely to dissolve charged solutes or solutes with high dipole moments (i.e., polar compounds) [199]. This is because the ability of a solvent to disrupt solute-solute interactions depends on the specific intermolecular forces involved.

One parameter used to estimate solvent polarity is the zero-frequency component of the dielectric constant (ε). While other measures such as hydrogen bonding capacity, dipole moment, and acidity/basicity are also important, the dielectric constant remains a good rule of thumb for estimating the polarity of the solvent as well as how miscible solvents are with each other [200]. Formally, ε is the relative permittivity of a material compared to vacuum and is defined as the amount of polarization that a material will experience (i.e., the magnitude of dipole moments) when an electric field is applied to it [199,201]. This means that ε is a measure of the polarizability of a solvent, with solvents having ε ≳ 10–20 defined as polar. As points of reference, apolar organic solvents have a relatively low ε~2, while polar water has an ε~80 [202]. Solvents with similar dielectric constants are generally miscible.

Given the wide variety of chemistries thought necessary for the origin of life [203], it could have been possible for a variety of aqueous and nonaqueous media to contribute to the potential prebiotic reaction space. Polar solvents can be divided further into protic solvents (able to hydrogen bond or donate hydrogen) and aprotic solvents, which can be determined by looking at the solvent molecule’s structure.

Here, we report the ε of each of the prebiotic environments introduced above (Table 6).

### 3.8. Boiling, Melting/Freezing Temperatures

At higher pressures such as in the deep ocean [7], different phase transitions can occur such as the direct sublimation of ice to water vapor upon increasing temperature [218]. Additionally, hysteresis, such as in rock pores, has also been observed (i.e., the freezing temperature is not identical to the melting temperature [219]).

Knowing the phase transition temperatures of materials is important for several reasons. Phase transitions accessible to the temperatures and pressures on early Earth impact the abundance of solvents. Additionally, materials in different phases have very different properties. Carbon dioxide gas and supercritical liquid carbon dioxide will have different affinities for various prebiotically plausible chemicals [203,220,221], resulting in differences in the reactivities or plausible chemistries residing within such environments. Furthermore, it has been shown that freeze-thaw cycles in water could have contributed to primitive genetic biopolymer (i.e., RNA) replication and assembly [222,223].

Here, we report on the boiling (liquid to gas transition) and melting (solid to liquid transition) temperatures of each of the prebiotic environments introduced above at atmospheric pressure as a point of reference (Table 7). However, as there were different environments on early Earth with variable pressure such as near hydrothermal vent systems in the deep ocean [186], the temperatures reported here will change accordingly (and at pressures below the triple point, there may only be one phase-transition temperature, i.e., sublimation, physically possible). Additionally, for some systems such as condensed droplet microenvironments, “melting” may refer to the transition from the condensed phase to the uniform phase, as increasing temperatures will inhibit the non-covalent bonds required for the structure to form, and depends on the composition of the system [69,224,225].

### 3.9. Vapor Pressure

Vapor pressure is related to the volatility of a material (i.e., the amount of gas that is released from a material at any given point) with the boiling point being defined as when the vapor pressure of the liquid material is equivalent to the ambient pressure (Section 3.8). Extreme cases include the highly volatile ammonia [241] and non-volatile mineral oil [242]. Higher temperatures will result in higher vapor pressure as per the Antoine relation [243]. Vapor pressure is also applicable to solids that sublimate (e.g., dry ice) [244]. A related concept is Henry’s law for mixtures of gases, which relates the amount of a dissolved gas to the partial pressure of that gas.

The volatility of a prebiotic material impacts whether that reaction environment is stable at a given temperature, or whether it will spontaneously (and quickly) change form into a gas, even below the boiling point. For example, it has been reported that the vapor pressure of fatty acids [245] and fatty acid esters [246] decreases with increasing chain-length. This suggests that fatty acids on early Earth, which could undergo liquid-phase reactions at high temperatures such as in hot spring environments [6], may have been more biased toward longer-chain fatty acids, as shorter chain fatty acids would likely have been volatilized into the gas phase.

Here, we report on the vapor pressure of each of the prebiotic environments introduced above (Table 8).

### 3.10. Compressibility and Stiffness

**Figure 3:**
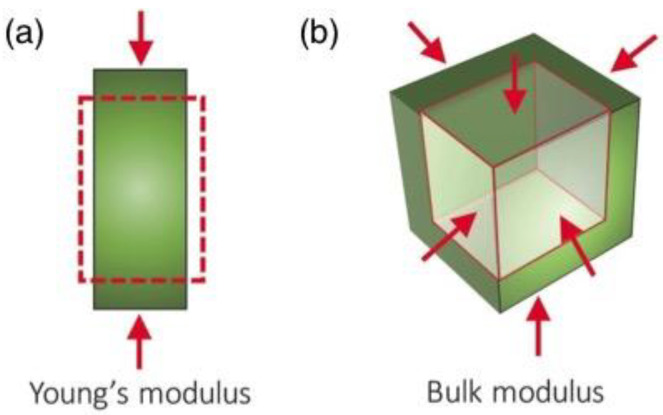
A physical description of the direction of forces used to calculate (**a**) the Young’s modulus (*E*) and (**b**) bulk modulus (*K*). Reprinted with permission from Burtch, NC, et al. “Mechanical Properties in Metal-Organic Frameworks: Emerging Opportunities and Challenges for Device Functionality and Technological Applications”. *Adv. Mater*. **30**(37), 1704124 (2018). [273] Copyright 2018 Wiley.

For solids, one measure of deformability is the Young’s Modulus (*E*) (Figure 3a). Materials with lower *E* are more compressible (less stiff), and vice versa. The *E* of coal is about 10 times less than limestone [255,256]. The Young’s modulus is only applicable to solids, as fluids (such as liquids and gases) require zero force to change in size lengthwise.

Bulk modulus (*K*) is defined as the amount of pressure (equally from all sides) required to effect a resulting volume change on a material (Figure 3b) and is defined for both fluids and solids. A typical gas has a very low *K* of <0.1 GPa [255] whereas quartz or clay has a *K* of 20–40 GPa [255].

While not immediately obvious, the compressibility and stiffness of materials become relevant on early Earth either in high-pressure environments, or when environments encounter high pressures resulting from a large external force. Examples include pressure from water deep in the ocean [186] or the late heavy bombardment during impacts [257]. The temporary deformation or compression of surface minerals with low *E*, for example, could have affected primitive mineral-driven processes such as mechanochemical sugar [258] or peptide [94,258] synthesis, possibly within mica sheets [259].

Here, we report the *K* and *E* of each of the prebiotic environments introduced above (Table 9).

### 3.11. Exposure to Radiation

Radiation is the process of energy transmission in the form of photons or massive particles and includes electromagnetic radiation (radio waves, microwaves, infrared, visible light, ultraviolet, X-rays, and gamma radiation) and particle radiation (e.g., alpha and beta particles, neutrons). Electromagnetic waves carry radiant energy as photons wherein the wavelength determines the energy, whereas particle radiation is the result of fast-moving subatomic particles whose energies can vary depending on their mass and velocities. Radiation is further classified as non-ionizing versus ionizing depending on the energy of the photon or particle. The energy of ionizing radiation is broadly defined to be between 10 and 33 eV, which is typically enough to ionize molecules and break chemical bonds (the energy of a C–C bond, for example, is about 3.6 eV). The region of electromagnetic radiation including higher energy ultraviolet, X-rays, and gamma radiation as well as typical particle radiation (alpha radiation, beta radiation and neutron radiation) are all considered types of ionizing radiation.

On early Earth, UV radiation would have been a strong driver of chemical synthesis and evolution. While the presence of ozone on modern Earth can absorb all ionizing and 98% of non-ionizing UV light, the surface of early Earth was exposed to much higher fluences of UV light (in particular, wavelengths longer than ~200 nm) prior to the build-up of atmospheric oxygen and thus ozone. In addition, while the young Sun was about 25% less luminous than today [274], its output in the UV region was likely larger. UV radiation has been demonstrated to play a key role in the synthesis of prebiotic molecules [275]. For example, the Sutherland group reported a UV radiation-driven photosynthesis of simple sugars from HCN [276], while UV radiation has also been shown to produce amino acids in the atmosphere [277]. However, UV radiation is also a double-edged sword, and has been shown to induce the degradation or structural/configurational alteration of organic molecules including DNA [278], proteins, and lipids [279].

Radiolysis from ionizing radiation is also employed in prebiotic chemistry as a synthetic mechanism [57]. Ionizing radiation could have been present through multiple sources such as cosmic rays and radioactive minerals. Cosmic rays consist of high-energy protons and atomic nuclei originating from the Sun or outside of the Solar System [280]; the energy spectrum of primary cosmic rays is between 10^16^ eV (2.31 × 10^17^ kcal/mol or 3.89 × 10^17^ kT, at 298 K) and 10^18^ eV (2.31 × 10^19^ kcal/mol or 3.89 × 10^19^ kT, at 298 K) [281]. Cosmic rays impact Earth’s upper atmosphere to produce showers of secondary photons and particles. A higher output of solar energetic particles from the young Sun via more frequent solar flares and coronal mass ejections has been proposed to have exposed the atmosphere of the early Earth to significantly higher fluxes of radiation in comparison to today [282]. Ancient radioactive mineral deposits containing, for example, monazite and uraninite [283], could also have provided locally high fluxes of alpha, beta, or gamma radiation on their surfaces [284]. Under the primordial conditions of the Hadean eon when the ^235^U isotope was much more abundant, so-called natural nuclear reactors that can promote self-sustaining nuclear fission chain reactions may have been relatively commonplace and produced highly radioactive local environments [285]. Over a dozen individual fission zones are known from the Oklo locality in Gabon [283]. A typical fission zone comparable in size to those found at Oklo are thought to produce ~10 kilowatts of power output (radiolysis and heat) wherein ~13% of this power is composed of γ or β rays that can penetrate substantial distances beyond their host minerals [283,284].

The availability of radiation in prebiotic reaction environments is determined by how far the radiation is able to penetrate a given material and the characteristics of the radiation source. Gamma radiation, arising from the radioactive decay of atomic nuclei, is characterized by short-wavelength electromagnetic waves (~10^–11^ m) with the highest photon energies above 100 keV. Gamma radiation is capable of penetrating materials to significant depths, requiring thick layers of high-density materials to block it. In comparison, alpha radiation with a fast-moving helium-4 nucleus is halted by a sheet of paper, and beta radiation consisting of high-velocity electrons can be stopped by an aluminum plate. The spectrum of the young Sun and attenuation of UV light by gases and aqueous media is covered thoroughly by Ranjan and Sasselov [275].

## 4. Conclusions

Here, we introduced some important physicochemical properties of various prebiotic reaction environments, with some brief examples of relevant prebiotic processes that could have been modulated by those physicochemical properties. Correlating physicochemical properties with reaction environments, while considering such properties during the design of prebiotically plausible reactions is highly relevant to the origins of life field. However, values of physicochemical characteristics in some environments still remain to be elucidated. This work serves as a request for the community to contribute to “filling in the blanks” in future works, so that researchers in the field can have a more holistic understanding of the prebiotic geochemical reaction environment. We also acknowledge the fact that experiments that fill in these blanks could be rather tedious and may not lead to high-profile publications; however, such studies are essential to pursue. 

From this overview, it is evident that an extremely wide range of physicochemical conditions can be accessed through the early Earth environment, which further supports the widely accepted notion that a large repertoire of chemical reactions were taking place on early Earth. Likewise, the prebiotic chemical repertoire is also highly diverse, with reactions or processes that may have had a range of tolerances to a wide variety of conditions and others that did not. The fact that modern biology, especially in the form of extremophiles, is also tolerant to a wide variety of conditions could be an artifact of the chemical reactions or processes that led to the life’s origins.

Generally speaking, the robustness/tolerance of the entire prebiotic chemical reaction repertoire to changes in conditions is at present not well-understood due to the large parameter space. In future, probing the “limits” of a wide range of prebiotic processes is necessary to gain a better understanding of which prebiotic environments could have plausibly hosted certain prebiotic chemical reaction networks. By aggregating this type of data, it will also be possible to conjecture which groups of prebiotic chemical processes/reactions could have been co-localized with each other as well as those which likely could not have occurred simultaneously under the same conditions in the same location.

The environments, properties, and examples of prebiotic processes provided here are but a brief and general overview of the entire prebiotic chemical milieu, and are not meant to be an exhaustive or comprehensive resource. A number of parameters for many primitive environments are not known (and as such, cannot be presented here), which behooves the field to continue characterizing the unknown physicochemical properties of all reaction environments. We further look forward to the field’s continuing exploration of extraterrestrial reaction environments, which could also provide insights into the possible reaction conditions on early Earth.

## Figures and Tables

**Table 1:** The ionic strength of different reaction environments.

Environment	Ionic Strength Range (M)	References
Aqueous solution	0.1–0.8 (oceans) 0.002–6 (lakes) 0.1–17 (lagoons) 0.1–7 (seas) 0.7–6 (hydrothermal brines)	[105]
Sea spray	Up to 6 (marine aerosol)	[106]
Gels	Variable, depending on components. Salt can dramatically alter gel properties.	[107,108]
Deep eutectic solvents	Ranges from 0 to >1, but ionic strength may not be the relevant principle.	[109,110]
Pure formamide	0.024 (commercially available pure formamide contains a significant amount of ionic impurities)	[111]
Lipid bilayer vesicle lumens (interior)	0–0.6, depends on the solution in which the amphiphilic molecules self-assemble.	[112,113]
Condensed droplet microenvironments	Up to 15 (within coacervate droplets)	[79]
Solid mineral surfaces	No ionic strength for solid mineral surfaces, surface charge density may be the more relevant parameter.	

**Table 2:** The viscosity of different reaction environments.

Environment	Typical Viscosity (mPas)	References
Aqueous solution	0.89–1.00 (freshwater at room temperature) Up to 1.3 (seawater at room temperature, depending on salinity)	[123,124,125]
Sea spray	Ranges from 1 (sea water) to 10–10,000 during evaporation or in presence of organics	[126]
Gels	Ranges from 1 up to 2 × 10^6^ (colloidal silica gel)	[127]
Ice	10^15^	[128]
Deep eutectic solvents	Variable; >100 and as high as 1700 possible	[129,130]
Formamide	3.23	[131]
High pressure supercritical fluids	0.02–0.16 (CO_2_, depending on pressure) 2.98 (water)	[132,133,134]
Tars	10–over 10^10^	[135]
Inside lipid bilayers	2D diffusion ~100–1000 1–1500 (heterogeneous)	[136]
Condensed droplet microenvironments	100 (coacervate)	[137]
Solid mineral surfaces	<1.0 × 10^28^ (crust)	[138]
Mantle	2.8 × 10^25^	[138]

**Table 3:** The specific heat of different reaction environments.

Environment	Specific Heat (kJ/Kg K)	References
Aqueous solution	4.18 (freshwater) 3.6–4.18 (saltwater), at room temperature.	[124,125,145,146]
Sea spray	Aerosols readily evaporate; specific heat is not very relevant.	
Gels	0.8–1.10 (silica gel) Specific heat for hydrogels depends on water level and temperature, for example, up to 30.	[147,148]
Ice	0.4873–0.3496 (from 0 to −80 °C, respectively)	[149]
Deep eutectic solvent	1.5–1.8 (example of salt eutectic)	[150]
Formamide	2.39	[151]
High pressure supercritical fluids	3–30 (CO_2_, depending on pressure) 27–690 (water, depending on pressure)	[152,153]
Tars	1.25–2	[154]
Inside bilayers	0.3–0.9; higher near melting temperature	[155,156]
Condensed droplet microenvironments	1.483	[157]
Solid mineral surfaces	0.180 (bromyrite) to 1.510 (epsomite); however, most are between 0.3 and 0.9	[158]
Mantle	1.250	[159]

**Table 4:** The pH range of different reaction environments.

Environment	pH Range	References
Aqueous solution	6.3–7.2 (4.0 Ga ocean)	[4,166,167,168]
	6.5–7.7 (2.5 Ga ocean)	
	8.2 (modern ocean)	
	6–8 (freshwater)	
	Pure water is 7.0	
	Hot spring environments have more variability, and can range from very acidic (less than pH 3) to somewhat alkaline (as high as pH 10).	
Sea spray	Around 8.0	[169]
Gels	Variable, depending on components.	[75,170,171,172,173]
Deep eutectic solvents	1.2–13.5 (eutectic at room temperature; pH varies greatly between eutectics, and also changes with temperature, down to pH 0)	[174,175]
High pressure supercritical fluids	2.80–2.95 (of water around scCO_2_)	[176]
Inside lipid bilayers	pH can be of a variety of ranges such as low as pH 2 or lower [177] or as high as pH 12 [178].	
Condensed droplet microenvironments	Highly dependent on the components, and especially their charge states at different pH (i.e., pKa).	
Solid mineral surfaces	Aqueous solutions containing solid mineral surfaces are mostly acidic. However, some have been found that were alkaline (pH 8.7–9.6).	[179]
Mantle	Mantle-derived igneous rocks can be alkaline, while mantle-derived minerals on the seafloor (around hydrothermal systems) can be around pH 9–11	[180,181]

**Table 5:** The density of the different reaction environments.

Environment	Density (g/mL)	References
Aqueous solution	0.9999749 (freshwater at 4 °C); 0.9970470 (freshwater at 25 °C) 1.025 (seawater, average; can be up to 1.09 depending on salinity)	[124,125,187]
Sea spray	1.12–2.16 (at room temperature)	[188,189]
Gels	Lower bound is that of the solvent for dilute gels.	
Ice	0.84–0.91 (sea ice)	[190]
Deep eutectic solvent	0.8–1.8 (example of a eutectic between 5 and 100 °C)	[174,191]
Formamide	1.129 (at 25 °C)	[131]
High pressure supercritical fluids	0.1–1 (CO_2_, depending on temperature and pressure) ~0.1–0.326 (water, depending on temperature and pressure)	[134,192,193]
Tars	1.1–1.23	[194,195]
Lipid bilayers	~0.9 for the lipid bilayer itself (e.g., decanoic acid density is 0.893 g/cm^3^) In the aqueous lumen, values as per ‘aqueous solution’.	[196]
Condensed droplet microenvironments	1.18–1.92	[197]
Solid mineral surfaces	1.2 (kerogen) to 10.969 (uraninite); however, most are typically between 2 and 7	[158,198]
Mantle	3.4 (mantle surface, and gets larger deeper)	[198]

**Table 6:** The dielectric constant (ε) of different reaction environments.

Environment	ε (unitless)	References
Aqueous solution	~70–80 (decreases with increasing temperature and salinity; seawater may be slightly lower than freshwater)	[204,205]
Sea spray	2.5–50	[206]
Gels	1.008–1.9 (silica gel, depending on density)	[207]
Ice	30–130 (ice)	[208]
Deep eutectic solvent	22.8 (one example)	[109]
Formamide	105–113 (room temperature)	[209,210]
High pressure supercritical fluids	1.07–1.46 (CO_2_, depending on temperature and pressure)	[211]
Tars	Up to 8 (coal tar)	[195,212]
Inside bilayers	2–3, can be higher for membranes that are more permeable than phospholipids	[213,214]
Condensed droplet microenvironments	40–50	[215]
Solid mineral surfaces	4.9–7.5	[216]
Mantle	~38 (water in the upper mantle at 300 km and 1000 K)	[217]

**Table 7:** The boiling and melting/freezing temperatures of different reaction environments.

Environment	Boiling Temperature	Melting/Freezing Temperature	References
Aqueous solution	Freshwater (100 °C); As high as 102 °C (seawater, depending on salinity)	Freshwater (0 °C); As low as –2 °C (seawater, depending on salinity)	[124,125,226,227,228]
Sea spray	70–100 °C	Close to 0 °C	[229,230]
Gels	2230 °C (silica gel)	1710 °C (silica gel)	[231]
Ice (eutectic)	In solid form, same as water (depending on salinity).		
Formamide	210 °C	2–3 °C	[232,233]
High pressure supercritical fluids	See footnote *		
Tars	190–400 °C		[234]
Inside lipid bilayers	See footnote ^		
Solid mineral surfaces	N/A	700–900 °C	[235]
Mantle	N/A	~3600 °C near the core–mantle boundary	[236]

* Typical “boiling” and “melting/freezing” transitions may not be applicable. Rather, the supercritical fluid to liquid, solid, and/or gas transition temperatures will depend on the pressure and is unique to each system based on the phase diagram. For example, scCO_2_ will transition to the liquid state below 304 K at 100 bar, but will transition to the solid state below 304 K at 10,000 bar [220]. Supercritical water will transition to the liquid state below 647 K at any pressure above 22.1 MPa; supercritical water cannot directly transition to the solid form under any circumstances [237]. Neither scCO_2_ nor supercritical water can transition to the gas phase based on temperature changes and can only transition to the gas phase upon decreasing pressure. ^ The lipid bilayer itself may not boil (as boiling requires the bilayer to vaporize, effectively resulting in the loss of the bilayer structure). However, the boiling point of the lipids that compose the bilayer vary depending on lipid composition. Typically, the boiling point increases with an increasing chain length; for example, caproic acid (C6 saturated) has a boiling point of 205.8 °C, while stearic acid (C18 saturated) has a boiling point of 376.1 °C [238]. The “melting” of a bilayer refers to the solid (gel) to liquid transition, and not the melting of the lipid components themselves. This also depends on the lipid composition; longer chain lipids typically have a higher phase transition temperature [239,240].

**Table 8:** The vapor pressure of different reaction environments.

Environment	Vapor Pressure (kPa)	References
Aqueous solution	2.3–4.2 (freshwater, room temperature) 2.1–3.9 (seawater, room temperature, depending on salinity)	[124,125]
Gels	~0.13–2.3, depending on the gel formulation and conditions.	[247]
Ice	6.1 (ice at 0 °C), but decreases with decreasing temperature (for example, 0.1 at −20 °C and 0.0014 at −100 °C).	[248]
Deep eutectic solvent	1.48 (CaCl_2_ eutectic in water at 20 °C). However, vapor pressure of other eutectics may vary depending on composition and temperature.	[249,250]
Formamide	0.008	[233]
Inside bilayers	Vapor pressure will be related to the vapor pressure of the bilayer components; vapor pressure typically decreases with increasing chain length (at constant temperature).	[251,252]
Condensed droplet microenvironments	Very low to negligible vapor pressure (ionic liquids)	[253]
Solid mineral surfaces	Around 0.05–0.25 (melted minerals >1900 K) Vapor pressure of solid mineral surfaces is negligible	[254]

**Table 9:** The Young’s (*E*) and bulk (*K*) modulus of different reaction environments.

Environment	*E* (GPa) *	*K* (GPa)	References
Aqueous solution	-	2.1	[260]
Gels	0.05–10 (of a silica aerogel, depending on gel density)	4–20 (of an alkaline-calcium silica hydrogel, depending on pressure)	[261,262]
Ice	8.6–12 (depends on the plane)	8.5–11.5 (depends on temperature)	[263,264]
High pressure supercritical fluids	-	1 (water at room temperature and pressure) 0.1–0.7 (CO_2_, depending on temperature and pressure)	[192,265,266]
Inside bilayers	0.02–0.03	0.6–0.9 (depending on temperature and location)	[267,268,269]
Condensed droplet microenvironments	These values will all depend on the droplet composition; “aging” is also an issue in these droplets.		
Solid mineral surfaces	6.38–288 (depending on the mineral and pressure)	40–120 (depending on mineral and pressure)	[270,271,272]
Mantle	150–720 (depending on depth)	100–600 (depending on depth)	[272]

* The Young’s modulus is not defined for liquids and gases.

## Data Availability

No new data was generated by this study.

## References

[1] Cleaves H.J. (2013). Prebiotic Chemistry: Geochemical Context and Reaction Screening. Life.

[2] Barge L.M. (2018). Considering Planetary Environments in Origin of Life Studies. Nat. Commun..

[3] Lunine J.I. (2006). Physical Conditions on the Early Earth. Philos. Trans. R. Soc. Lond. B Biol. Sci..

[4] Krissansen-Totton J., Arney G.N., Catling D.C. (2018). Constraining the Climate and Ocean pH of the Early Earth with a Geological Carbon Cycle Model. Proc. Natl. Acad. Sci. USA.

[5] Van Kranendonk M.J. (2014). Earth’s Early Atmosphere and Surface Environments: A Review. Earth’s Early Atmosphere and Surface Environment.

[6] Damer B., Deamer D. (2020). The Hot Spring Hypothesis for an Origin of Life. Astrobiology.

[7] Zhang X., Li L.-F., Du Z.-F., Hao X.-L., Cao L., Luan Z.-D., Wang B., Xi S.-C., Lian C., Yan J. (2020). Discovery of Supercritical Carbon Dioxide in a Hydrothermal System. Sci. Bull..

[8] Balucani N. (2009). Elementary Reactions and Their Role in Gas-Phase Prebiotic Chemistry. Int. J. Mol. Sci..

[9] Rimmer P.B., Shorttle O. (2019). Origin of Life’s Building Blocks in Carbon- and Nitrogen-Rich Surface Hydrothermal Vents. Life.

[10] Green N.J., Xu J., Sutherland J.D. (2021). Illuminating Life’s Origins: UV Photochemistry in Abiotic Synthesis of Biomolecules. J. Am. Chem. Soc..

[11] Cooper G.M. (2000). Cell Membranes. The Cell: A Molecular Approach.

[12] Ghosh B., Bose R., Tang T.-Y.D. (2021). Can Coacervation Unify Disparate Hypotheses in the Origin of Cellular Life?. Curr. Opin. Colloid Interface Sci..

[13] Yoshizawa T., Nozawa R.-S., Jia T.Z., Saio T., Mori E. (2020). Biological Phase Separation: Cell Biology Meets Biophysics. Biophys. Rev..

[14] Hayes A.G. (2016). The Lakes and Seas of Titan. Annu. Rev. Earth Planet. Sci..

[15] Barnes J.W., Turtle E.P., Trainer M.G., Lorenz R.D., MacKenzie S.M., Brinckerhoff W.B., Cable M.L., Ernst C.M., Freissinet C., Hand K.P. (2021). Science Goals and Objectives for the Dragonfly Titan Rotorcraft Relocatable Lander. Planet. Sci. J..

[16] Pizzarello S., Shock E. (2010). The Organic Composition of Carbonaceous Meteorites: The Evolutionary Story ahead of Biochemistry. Cold Spring Harb. Perspect. Biol..

[17] Arumainayagam C.R., Garrod R.T., Boyer M.C., Hay A.K., Bao S.T., Campbell J.S., Wang J., Nowak C.M., Arumainayagam M.R., Hodge P.J. (2019). Extraterrestrial Prebiotic Molecules: Photochemistry vs. Radiation Chemistry of Interstellar Ices. Chem. Soc. Rev..

[18] Van Dishoeck E.F., Herbst E., Neufeld D.A. (2013). Interstellar Water Chemistry: From Laboratory to Observations. Chem. Rev..

[19] Lasne J., Noblet A., Szopa C., Navarro-González R., Cabane M., Poch O., Stalport F., François P., Atreya S.K., Coll P. (2016). Oxidants at the Surface of Mars: A Review in Light of Recent Exploration Results. Astrobiology.

[20] Deamer D., Damer B. (2017). Can Life Begin on Enceladus? A Perspective from Hydrothermal Chemistry. Astrobiology.

[21] Cable M.L., Hörst S.M., Hodyss R., Beauchamp P.M., Smith M.A., Willis P.A. (2012). Titan Tholins: Simulating Titan Organic Chemistry in the Cassini-Huygens Era. Chem. Rev..

[22] Kitadai N., Maruyama S. (2018). Origins of Building Blocks of Life: A Review. Geosci. Front..

[23] Cleaves H.J. (2012). Prebiotic Chemistry: What We Know, What We Don’t. Evolution.

[24] Ruiz-Mirazo K., Briones C., de la Escosura A. (2014). Prebiotic Systems Chemistry: New Perspectives for the Origins of Life. Chem. Rev..

[25] Deamer D., Singaram S., Rajamani S., Kompanichenko V., Guggenheim S. (2006). Self-Assembly Processes in the Prebiotic Environment. Philos. Trans. R. Soc. Lond. B Biol. Sci..

[26] Tanford C. (1978). The Hydrophobic Effect and the Organization of Living Matter. Science.

[27] Das S., Lin Y.-H., Vernon R.M., Forman-Kay J.D., Chan H.S. (2020). Comparative Roles of Charge, and Hydrophobic Interactions in Sequence-Dependent Phase Separation of Intrinsically Disordered Proteins. Proc. Natl. Acad. Sci. USA.

[28] Maibaum L., Dinner A.R., Chandler D. (2004). Micelle Formation and the Hydrophobic Effect. J. Phys. Chem. B.

[29] Farmer J.R., Sigman D.M., Granger J., Underwood O.M., Fripiat F., Cronin T.M., Martínez-García A., Haug G.H. (2021). Arctic Ocean Stratification Set by Sea Level and Freshwater Inputs since the Last Ice Age. Nat. Geosci..

[30] Mast C.B., Schink S., Gerland U., Braun D. (2013). Escalation of Polymerization in a Thermal Gradient. Proc. Natl. Acad. Sci. USA.

[31] Herschy B., Whicher A., Camprubi E., Watson C., Dartnell L., Ward J., Evans J.R.G., Lane N. (2014). An Origin-of-Life Reactor to Simulate Alkaline Hydrothermal Vents. J. Mol. Evol..

[32] Kreysing M., Keil L., Lanzmich S., Braun D. (2015). Heat Flux across an Open Pore Enables the Continuous Replication and Selection of Oligonucleotides towards Increasing Length. Nat. Chem..

[33] Dobson C.M., Ellison G.B., Tuck A.F., Vaida V. (2000). Atmospheric Aerosols as Prebiotic Chemical Reactors. Proc. Natl. Acad. Sci. USA.

[34] Donaldson D.J., Tervahattu H., Tuck A.F., Vaida V. (2004). Organic Aerosols and the Origin of Life: An Hypothesis. Orig. Life Evol. Biosph..

[35] Rapf R.J., Perkins R.J., Dooley M.R., Kroll J.A., Carpenter B.K., Vaida V. (2018). Environmental Processing of Lipids Driven by Aqueous Photochemistry of α-Keto Acids. ACS Cent. Sci..

[36] Fournier R.O., Rowe J.J. (1966). The Deposition of Silica in Hot Springs. Bull. Volcanol..

[37] Mamajanov I. (2019). Wet-Dry Cycling Delays the Gelation of Hyperbranched Polyesters: Implications to the Origin of Life. Life.

[38] Dass A.V., Jaber M., Brack A., Foucher F., Kee T.P., Georgelin T., Westall F. (2018). Potential Role of Inorganic Confined Environments in Prebiotic Phosphorylation. Life.

[39] Campbell T.D., Febrian R., McCarthy J.T., Kleinschmidt H.E., Forsythe J.G., Bracher P.J. (2019). Prebiotic Condensation through Wet–dry Cycling Regulated by Deliquescence. Nat. Commun..

[40] Mamajanov I., Caudan M., Jia T.Z. (2020). Protoenzymes: The Case of Hyperbranched Polymer-Scaffolded ZnS Nanocrystals. Life.

[41] Nguyen V.S., Orlando T.M., Leszczynski J., Nguyen M.T. (2013). Theoretical Study of the Decomposition of Formamide in the Presence of Water Molecules. J. Phys. Chem. A.

[42] Bizzarri B.M., Saladino R., Delfino I., García-Ruiz J.M., Di Mauro E. (2021). Prebiotic Organic Chemistry of Formamide and the Origin of Life in Planetary Conditions: What We Know and What Is the Future. Int. J. Mol. Sci..

[43] Saitta A.M., Saija F. (2014). Miller Experiments in Atomistic Computer Simulations. Proc. Natl. Acad. Sci. USA.

[44] Koike T., Kaneko T., Kobayashi K., Miyakawa S., Takano Y. (2003). Formation of Organic Compounds from Simulated Titan Atmosphere: Perspectives of the Cassini Mission. Biol. Sci. Space.

[45] Gerakines P.A., Moore M.H., Hudson R.L. (2004). Ultraviolet Photolysis and Proton Irradiation of Astrophysical Ice Analogs Containing Hydrogen Cyanide. Icarus.

[46] Takano Y., Tsuboi T., Kaneko T., Kobayashi K., Marumo K. (2004). Pyrolysis of High-Molecular-Weight Complex Organics Synthesized from a Simulated Interstellar Gas Mixture Irradiated with 3 MeV Proton Beam. Bull. Chem. Soc. Jpn..

[47] Saladino R., Crestini C., Pino S., Costanzo G., Di Mauro E. (2012). Formamide and the Origin of Life. Phys. Life Rev..

[48] He C., Gállego I., Laughlin B., Grover M.A., Hud N.V. (2017). A Viscous Solvent Enables Information Transfer from Gene-Length Nucleic Acids in a Model Prebiotic Replication Cycle. Nat. Chem..

[49] Abbott A.P., Capper G., Davies D.L., Rasheed R.K., Tambyrajah V. (2003). Novel Solvent Properties of Choline Chloride/urea Mixtures. Chem. Commun..

[50] Hansen B.B., Spittle S., Chen B., Poe D., Zhang Y., Klein J.M., Horton A., Adhikari L., Zelovich T., Doherty B.W. (2021). Deep Eutectic Solvents: A Review of Fundamentals and Applications. Chem. Rev..

[51] Gull M., Zhou M., Fernández F.M., Pasek M.A. (2014). Prebiotic Phosphate Ester Syntheses in a Deep Eutectic Solvent. J. Mol. Evol..

[52] Chien C.-Y., Yu S.-S. (2020). Ester-Mediated Peptide Formation Promoted by Deep Eutectic Solvents: A Facile Pathway to Proto-Peptides. Chem. Commun..

[53] Smith E.L., Abbott A.P., Ryder K.S. (2014). Deep Eutectic Solvents (DESs) and Their Applications. Chem. Rev..

[54] Smith P.J., Arroyo C.B., Lopez Hernandez F., Goeltz J.C. (2019). Ternary Deep Eutectic Solvent Behavior of Water and Urea? Choline Chloride Mixtures. J. Phys. Chem. B.

[55] Jiménez-Serra I., Martín-Pintado J., Rivilla V.M., Rodríguez-Almeida L., Alonso Alonso E.R., Zeng S., Cocinero E.J., Martín S., Requena-Torres M., Martín-Domenech R. (2020). Toward the RNA-World in the Interstellar Medium-Detection of Urea and Search of 2-Amino-Oxazole and Simple Sugars. Astrobiology.

[56] Kaiser R.I., Maity S., Jones B.M. (2015). Synthesis of Prebiotic Glycerol in Interstellar Ices. Angew. Chem. Int. Ed. Engl..

[57] Yi R., Tran Q.P., Ali S., Yoda I., Adam Z.R., Cleaves H.J., Fahrenbach A.C. (2020). A Continuous Reaction Network That Produces RNA Precursors. Proc. Natl. Acad. Sci. USA.

[58] Raveendran P., Ikushima Y., Wallen S.L. (2005). Polar Attributes of Supercritical Carbon Dioxide. Acc. Chem. Res..

[59] Deguchi S., Tsujii K. (2007). Supercritical Water: A Fascinating Medium for Soft Matter. Soft Matter.

[60] Guttenberg N., Virgo N., Chandru K., Scharf C., Mamajanov I. (2017). Bulk Measurements of Messy Chemistries Are Needed for a Theory of the Origins of Life. Philos. Trans. R. Soc. A.

[61] Barranco F.T., Dawson H.E. (1999). Influence of Aqueous pH on the Interfacial Properties of Coal Tar. Environ. Sci. Technol..

[62] Serrano-Luginbühl S., Ruiz-Mirazo K., Ostaszewski R., Gallou F., Walde P. (2018). Soft and Dispersed Interface-Rich Aqueous Systems That Promote and Guide Chemical Reactions. Nat. Rev. Chem..

[63] Israelachvili J.N. (2010). Intermolecular and Surface Forces.

[64] Hanczyc M.M., Mansy S.S., Szostak J.W. (2007). Mineral Surface Directed Membrane Assembly. Orig. Life Evol. Biosph..

[65] Dey A., Bomans P.H.H., Müller F.A., Will J., Frederik P.M., de With G., Sommerdijk N.A.J.M. (2010). The Role of Prenucleation Clusters in Surface-Induced Calcium Phosphate Crystallization. Nat. Mater..

[66] Köksal E.S., Liese S., Kantarci I., Olsson R., Carlson A., Gözen I. (2019). Nanotube-Mediated Path to Protocell Formation. ACS Nano.

[67] Steller L.H., Van Kranendonk M.J., Wang A. (2022). Dehydration Enhances Prebiotic Lipid Remodeling and Vesicle Formation in Acidic Environments. ACS Cent. Sci..

[68] Rajamani S., Vlassov A., Benner S., Coombs A., Olasagasti F., Deamer D. (2008). Lipid-Assisted Synthesis of RNA-like Polymers from Mononucleotides. Orig. Life Evol. Biosph..

[69] Fraccia T.P., Jia T.Z. (2020). Liquid Crystal Coacervates Composed of Short Double-Stranded DNA and Cationic Peptides. ACS Nano.

[70] Poudyal R.R., Pir Cakmak F., Keating C.D., Bevilacqua P.C. (2018). Physical Principles and Extant Biology Reveal Roles for RNA-Containing Membraneless Compartments in Origins of Life Chemistry. Biochemistry.

[71] Koga S., Williams D.S., Perriman A.W., Mann S. (2011). Peptide-Nucleotide Microdroplets as a Step towards a Membrane-Free Protocell Model. Nat. Chem..

[72] Cakmak F.P., Keating C.D. (2017). Combining Catalytic Microparticles with Droplets Formed by Phase Coexistence: Adsorption and Activity of Natural Clays at the Aqueous/Aqueous Interface. Sci. Rep..

[73] Guo W., Kinghorn A.B., Zhang Y., Li Q., Poonam A.D., Tanner J.A., Shum H.C. (2021). Non-Associative Phase Separation in an Evaporating Droplet as a Model for Prebiotic Compartmentalization. Nat. Commun..

[74] Jia T.Z., Hentrich C., Szostak J.W. (2014). Rapid RNA Exchange in Aqueous Two-Phase System and Coacervate Droplets. Orig. Life Evol. Biosph..

[75] Jia T.Z., Chandru K., Hongo Y., Afrin R., Usui T., Myojo K., Cleaves H.J. (2019). Membraneless Polyester Microdroplets as Primordial Compartments at the Origins of Life. Proc. Natl. Acad. Sci. USA.

[76] Jia T.Z., Bapat N.V., Verma A., Mamajanov I., Cleaves H.J., Chandru K. (2021). Incorporation of Basic α-Hydroxy Acid Residues into Primitive Polyester Microdroplets for RNA Segregation. Biomacromolecules.

[77] Afrin R., Chen C., Sarpa D., Sithamparam M., Yi R., Giri C., Mamajanov I., Cleaves H.J., Chandru K., Jia T.Z. (2022). The Effects of Dehydration Temperature and Monomer Chirality on Primitive Polyester Synthesis and Microdroplet Assembly. Macromol. Chem. Phys..

[78] Mountain G.A., Keating C.D. (2020). Formation of Multiphase Complex Coacervates and Partitioning of Biomolecules within Them. Biomacromolecules.

[79] Frankel E.A., Bevilacqua P.C., Keating C.D. (2016). Polyamine/Nucleotide Coacervates Provide Strong Compartmentalization of Mg^2+^, Nucleotides, and RNA. Langmuir.

[80] Zaia D.A.M. (2004). A Review of Adsorption of Amino Acids on Minerals: Was It Important for Origin of Life?. Amino Acids.

[81] Pedreira-Segade U., Hao J., Razafitianamaharavo A., Pelletier M., Marry V., Le Crom S., Michot L.J., Daniel I. (2018). How Do Nucleotides Adsorb Onto Clays?. Life.

[82] Hashizume H. (2015). Adsorption of Nucleic Acid Bases, Ribose, and Phosphate by Some Clay Minerals. Life.

[83] Lambert J.-F. (2008). Adsorption and Polymerization of Amino Acids on Mineral Surfaces: A Review. Orig. Life Evol. Biosph..

[84] Erastova V., Degiacomi M.T., G Fraser D., Greenwell H.C. (2017). Mineral Surface Chemistry Control for Origin of Prebiotic Peptides. Nat. Commun..

[85] Rimola A., Sodupe M., Ugliengo P. (2019). Role of Mineral Surfaces in Prebiotic Chemical Evolution. In Silico Quantum Mechanical Studies. Life.

[86] Pérez-Aguilar C.D., Cuéllar-Cruz M. (2022). The Formation of Crystalline Minerals and Their Role in the Origin of Life on Earth. Prog. Cryst. Growth Charact. Mater..

[87] Gillams R.J., Jia T.Z. (2018). Mineral Surface-Templated Self-Assembling Systems: Case Studies from Nanoscience and Surface Science towards Origins of Life Research. Life.

[88] Cleaves H.J., Michalkova Scott A., Hill F.C., Leszczynski J., Sahai N., Hazen R. (2012). Mineral-Organic Interfacial Processes: Potential Roles in the Origins of Life. Chem. Soc. Rev..

[89] Hazen R.M. (2017). Chance, Necessity and the Origins of Life: A Physical Sciences Perspective. Philos. Trans. A Math. Phys. Eng. Sci..

[90] Li Y. (2022). Minerals as Prebiotic Catalysts for Chemical Evolution towards the Origin of Life. Mineralogy.

[91] Pasek M., Herschy B., Kee T.P. (2015). Phosphorus: A Case for Mineral-Organic Reactions in Prebiotic Chemistry. Orig. Life Evol. Biosph..

[92] Walton C.R., Shorttle O., Jenner F.E., Williams H.M., Golden J., Morrison S.M., Downs R.T., Zerkle A., Hazen R.M., Pasek M. (2021). Phosphorus Mineral Evolution and Prebiotic Chemistry: From Minerals to Microbes. Earth Sci. Rev..

[93] Gözen İ. (2021). Did Solid Surfaces Enable the Origin of Life?. Life.

[94] Stolar T., Grubešić S., Cindro N., Meštrović E., Užarević K., Hernández J.G. (2021). Mechanochemical Prebiotic Peptide Bond Formation*. Angew. Chem. Int. Ed. Engl..

[95] Cleaves H.J., Chalmers J.H., Lazcano A., Miller S.L., Bada J.L. (2008). A Reassessment of Prebiotic Organic Synthesis in Neutral Planetary Atmospheres. Orig. Life Evol. Biosph..

[96] Ogawa M. (2008). Mantle Convection: A Review. Fluid Dyn. Res..

[97] Frost D.J., Liebske C., Langenhorst F., McCammon C.A., Trønnes R.G., Rubie D.C. (2004). Experimental Evidence for the Existence of Iron-Rich Metal in the Earth’s Lower Mantle. Nature.

[98] Benner S.A., Kim H.-J., Biondi E. (2019). Prebiotic Chemistry That Could Not Not Have Happened. Life.

[99] Labrosse S., Hernlund J.W., Coltice N. (2007). A Crystallizing Dense Magma Ocean at the Base of the Earth’s Mantle. Nature.

[100] Hu Q., Kim D.Y., Liu J., Meng Y., Yang L., Zhang D., Mao W.L., Mao H.-K. (2017). Dehydrogenation of Goethite in Earth’s Deep Lower Mantle. Proc. Natl. Acad. Sci. USA.

[101] Sastre de Vicente M.E. (2004). The Concept of Ionic Strength Eighty Years after Its Introduction in Chemistry. J. Chem. Educ..

[102] Khouri S.J. (2015). Titrimetric Study of the Solubility and Dissociation of Benzoic Acid in Water: Effect of Ionic Strength and Temperature. Am. J. Analyt. Chem..

[103] Hu Y., Li K., Li Y., Liu H., Guo M., Ye X., Wu Z., Lee K. (2019). Dyes Adsorption onto Fe_3_O_4_ -bis(trimethoxysilylpropyl)amine Composite Particles: Effects of pH and Ionic Strength on Electrostatic Interactions. ChemistrySelect.

[104] Melkikh A.V., Sutormina M.I. (2008). Model of Active Transport of Ions in Cardiac Cell. J. Theor. Biol..

[105] Millero F.J. (1985). The Physical Chemistry of Natural Waters. Pure Appl. Chem..

[106] Mekic M., Gligorovski S. (2021). Ionic Strength Effects on Heterogeneous and Multiphase Chemistry: Clouds versus Aerosol Particles. Atmos. Environ..

[107] Feng Y., Taraban M., Yu Y.B. (2012). The Effect of Ionic Strength on the Mechanical, Structural and Transport Properties of Peptide Hydrogels. Soft Matter.

[108] Anderson M.A., Tomić M., Gieselmann M.J., Villegas M.A. (1989). The Critical Gelling Point in Silica Gels Containing Lithium, Sodium and Potassium. J. Non-Cryst. Solids.

[109] Sanchez-Fernandez A., Jackson A.J., Prévost S.F., Doutch J.J., Edler K.J. (2021). Long-Range Electrostatic Colloidal Interactions and Specific Ion Effects in Deep Eutectic Solvents. J. Am. Chem. Soc..

[110] Abbott A.P., Edler K.J., Page A.J. (2021). Deep Eutectic Solvents-The Vital Link between Ionic Liquids and Ionic Solutions. J. Chem. Phys..

[111] Porras S.P., Kenndler E. (2004). Formamide as Solvent for Capillary Zone Electrophoresis. Electrophoresis.

[112] Redondo-Morata L., Oncins G., Sanz F. (2012). Force Spectroscopy Reveals the Effect of Different Ions in the Nanomechanical Behavior of Phospholipid Model Membranes: The Case of Potassium Cation. Biophys. J..

[113] Deamer D. (2017). The Role of Lipid Membranes in Life’s Origin. Life.

[114] Walton C.R., Shorttle O. (2021). Scum of the Earth: A Hypothesis for Prebiotic Multi-Compartmentalised Environments. Life.

[115] Huang Y., Barraza K.M., Kenseth C.M., Zhao R., Wang C., Beauchamp J.L., Seinfeld J.H. (2018). Probing the OH Oxidation of Pinonic Acid at the Air-Water Interface Using Field-Induced Droplet Ionization Mass Spectrometry (FIDI-MS). J. Phys. Chem. A.

[116] Morasch M., Liu J., Dirscherl C.F., Ianeselli A., Kühnlein A., Le Vay K., Schwintek P., Islam S., Corpinot M.K., Scheu B. (2019). Heated Gas Bubbles Enrich, Crystallize, Dry, Phosphorylate and Encapsulate Prebiotic Molecules. Nat. Chem..

[117] Hazen R.M., Sverjensky D.A. (2010). Mineral Surfaces, Geochemical Complexities, and the Origins of Life. Cold Spring Harb. Perspect. Biol..

[118] Gong J., Bao X. (2017). Fundamental Insights into Interfacial Catalysis. Chem. Soc. Rev..

[119] Scott D.L., White S.P., Otwinowski Z., Yuan W., Gelb M.H., Sigler P.B. (1990). Interfacial Catalysis: The Mechanism of Phospholipase A2. Science.

[120] Pera-Titus M., Leclercq L., Clacens J.-M., De Campo F., Nardello-Rataj V. (2015). Pickering Interfacial Catalysis for Biphasic Systems: From Emulsion Design to Green Reactions. Angew. Chem. Int. Ed. Engl..

[121] Carroll K.M., Knoll A.W., Wolf H., Duerig U. (2018). Explaining the Transition from Diffusion Limited to Reaction Limited Surface Assembly of Molecular Species through Spatial Variations. Langmuir.

[122] He C., Lozoya-Colinas A., Gállego I., Grover M.A., Hud N.V. (2019). Solvent Viscosity Facilitates Replication and Ribozyme Catalysis from an RNA Duplex in a Model Prebiotic Process. Nucleic Acids Res..

[123] Qasem N.A.A., Generous M.M., Qureshi B.A., Zubair S.M. (2021). A Comprehensive Review of Saline Water Correlations and Data: Part II—Thermophysical Properties. Arab. J. Sci. Eng..

[124] Nayar K.G., Sharqawy M.H., Banchik L.D., Lienhard J.H. (2016). Thermophysical Properties of Seawater: A Review and New Correlations That Include Pressure Dependence. Desalination.

[125] Sharqawy M.H., Lienhard J.H., Zubair S.M. (2010). Thermophysical Properties of Seawater: A Review of Existing Correlations and Data. Desalination Water Treat..

[126] Tumminello P.R., James R.C., Kruse S., Kawasaki A., Cooper A., Guadalupe-Diaz I., Zepeda K.L., Crocker D.R., Mayer K.J., Sauer J.S. (2021). Evolution of Sea Spray Aerosol Particle Phase State across a Phytoplankton Bloom. ACS Earth Space Chem..

[127] Ghaffari Z., Rezvani H., Khalilnezhad A., Cortes F.B., Riazi M. (2022). Experimental Characterization of Colloidal Silica Gel for Water Conformance Control in Oil Reservoirs. Sci. Rep..

[128] Fowler A.C. (1997). Glaciers and Ice Sheets. The Mathematics of Models for Climatology and Environment.

[129] Wang Y., Ma C., Liu C., Lu X., Feng X., Ji X. (2020). Thermodynamic Study of Choline Chloride-Based Deep Eutectic Solvents with Water and Methanol. J. Chem. Eng. Data.

[130] Lemaoui T., Darwish A.S., Attoui A., Abu Hatab F., Hammoudi N.E.H., Benguerba Y., Vega L.F., Alnashef I.M. (2020). Predicting the Density and Viscosity of Hydrophobic Eutectic Solvents: Towards the Development of Sustainable Solvents. Green Chem..

[131] Cases A.M., Gómez Marigliano A.C., Bonatti C.M., Sólimo H.N. (2001). Density, Viscosity, and Refractive Index of Formamide, Three Carboxylic Acids, and Formamide + Carboxylic Acid Binary Mixtures. J. Chem. Eng. Data.

[132] Heidaryan E., Hatami T., Rahimi M., Moghadasi J. (2011). Viscosity of Pure Carbon Dioxide at Supercritical Region: Measurement and Correlation Approach. J. Supercrit. Fluids.

[133] Deng J., Zhao G., Zhang L., Ma H., Song F., Cao Q., Zhang X. (2021). Simple and Accurate Calculation Model of Viscosity for Supercritical CO2. J. Phys. Conf. Ser..

[134] Zheng H., Yu T., Qu C., Li W., Wang Y. (2020). Basic Characteristics and Application Progress of Supercritical Water. IOP Conf. Ser. Earth Environ. Sci..

[135] Wood L.J., Downer M. (2007). Viscosity/temperature Equations for Coal Tar Pitches and Refined Tars. J. Appl. Chem..

[136] Nojima Y., Iwata K. (2014). Viscosity Heterogeneity inside Lipid Bilayers of Single-Component Phosphatidylcholine Liposomes Observed with Picosecond Time-Resolved Fluorescence Spectroscopy. J. Phys. Chem. B.

[137] Lu T., Spruijt E. (2020). Multiphase Complex Coacervate Droplets. J. Am. Chem. Soc..

[138] She Y., Fu G. (2019). Viscosities of the Crust and Upper Mantle Constrained by Three-Dimensional GPS Rates in the Sichuan–Yunnan Fragment of China. Earth Planets Space.

[139] Yousefi Y., Tariku F. (2021). Thermal Conductivity and Specific Heat Capacity of Insulation Materials at Different Mean Temperatures. J. Phys. Conf. Ser..

[140] Mallamace F., Corsaro C., Mallamace D., Fazio E., Chen S.-H., Cupane A. (2020). Specific Heat and Transport Functions of Water. Int. J. Mol. Sci..

[141] Waples D.W., Waples J.S. (2004). A Review and Evaluation of Specific Heat Capacities of Rocks, Minerals, and Subsurface Fluids. Part 2: Fluids and Porous Rocks. Nat. Resour. Res..

[142] Pirajno F. (2020). Subaerial Hot Springs and near-Surface Hydrothermal Mineral Systems Past and Present, and Possible Extraterrestrial Analogues. Geosci. Front..

[143] Kitadai N., Nakamura R., Yamamoto M., Okada S., Takahagi W., Nakano Y., Takahashi Y., Takai K., Oono Y. (2021). Thioester Synthesis through Geoelectrochemical CO_2_ Fixation on Ni Sulfides. Commun. Chem..

[144] Yang F., Wang G., Hu D., Zhou H., Tan X. (2022). Influence of Water-Rock Interaction on Permeability and Heat Conductivity of Granite under High Temperature and Pressure Conditions. Geothermics.

[145] Feistel R. (2003). A New Extended Gibbs Thermodynamic Potential of Seawater. Prog. Oceanogr..

[146] Millero F.J., Perron G., Desnoyers J.E. (1973). Heat Capacity of Seawater Solutions from 5° to 35 °C and 0.5 to 22‰ Chlorinity. J. Geophys. Res..

[147] Islam M.A., Pal A., Saha B.B. (2020). Experimental Study on Thermophysical and Porous Properties of Silica Gels. Int. J. Refrig..

[148] Di Maggio R., Dirè S., Callone E., Bergamonti L., Lottici P.P., Albatici R., Rigon R., Ataollahi N. (2020). Super-Adsorbent Polyacrylate under Swelling in Water for Passive Solar Control of Building Envelope. SN Appl. Sci..

[149] Barnes W.H., Maass O. (1930). Specific Heats and Latent Heat of Fusion of Ice. Can. J. Res..

[150] Seo J., Shin D. (2014). Enhancement of Specific Heat of Ternary Nitrate (LiNO_3_-NaNO_3_-KNO_3_) Salt by Doping with SiO_2_ Nanoparticles for Solar Thermal Energy Storage. Micro Nano Lett..

[151] De Wit H.G.M., De Kruif C.G., Van Miltenburg J.C. (1983). Thermodynamic Properties of Molecular Organic Crystals Containing Nitrogen, Oxygen, and Sulfur II. Molar Heat Capacities of Eight Compounds by Adiabatic Calorimetry. J. Chem. Thermodyn..

[152] Li W., Yu Z. (2021). Heat Exchangers for Cooling Supercritical Carbon Dioxide and Heat Transfer Enhancement: A Review and Assessment. Energy Rep..

[153] Pioro I., Mokry S. (2011). Thermophysical Properties at Critical and Supercritical Pressures. Heat Transfer-Theoretical Analysis, Experimental Investigations and Industrial Systems.

[154] Hyman D., Kay W.B. (1949). Heat Capacity and Content of Tars and Pitches. Ind. Eng. Chem..

[155] Marsh D. (2012). Thermodynamics of Phospholipid Self-Assembly. Biophys. J..

[156] Heimburg T. (1998). Mechanical Aspects of Membrane Thermodynamics. Estimation of the Mechanical Properties of Lipid Membranes close to the Chain Melting Transition from Calorimetry. Biochim. Biophys. Acta Biomembr..

[157] Yang P.H., Rupley J.A. (1979). Protein–Water Interactions. Heat Capacity of the Lysozyme–Water System. Biochemistry.

[158] Waples D.W., Waples J.S. (2004). A Review and Evaluation of Specific Heat Capacities of Rocks, Minerals, and Subsurface Fluids. Part 1: Minerals and Nonporous Rocks. Nat. Resour. Res..

[159] Jaupart C., Labrosse S., Lucazeau F., Mareschal J.-C. (2015). Temperatures, Heat, and Energy in the Mantle of the Earth. Treatise on Geophysics.

[160] Kahlert H., Leito I. (2019). Generalization of Acid-Base Diagrams Based on the Unified pH-Scale. Chemphyschem.

[161] Love C., Steinkühler J., Gonzales D.T., Yandrapalli N., Robinson T., Dimova R., Tang T.-Y.D. (2020). Reversible pH-Responsive Coacervate Formation in Lipid Vesicles Activates Dormant Enzymatic Reactions. Angew. Chem. Int. Ed. Engl..

[162] Mariani A., Bonfio C., Johnson C.M., Sutherland J.D. (2018). pH-Driven RNA Strand Separation under Prebiotically Plausible Conditions. Biochemistry.

[163] Rubio-Sánchez R., O’Flaherty D.K., Wang A., Coscia F., Petris G., Di Michele L., Cicuta P., Bonfio C. (2021). Thermally Driven Membrane Phase Transitions Enable Content Reshuffling in Primitive Cells. J. Am. Chem. Soc..

[164] Komatsu G., Senthil Kumar P., Goto K., Sekine Y., Giri C., Matsui T. (2014). Drainage Systems of Lonar Crater, India: Contributions to Lonar Lake Hydrology and Crater Degradation. Planet. Space Sci..

[165] Lee J., Kim G. (2015). Dependence of pH in Coastal Waters on the Adsorption of Protons onto Sediment Minerals. Limnol. Oceanogr..

[166] Dutta S., Sarma D., Nath P. (2015). Ground and River Water Quality Monitoring Using a Smartphone-Based pH Sensor. AIP Adv..

[167] Sasaki M. (2018). Classification of Water Types of Acid Hot-Spring Waters in Japan. J. Geotherm. Res. Soc. Jpn..

[168] Poddar A., Das S.K. (2018). Microbiological Studies of Hot Springs in India: A Review. Arch. Microbiol..

[169] Angle K.J., Crocker D.R., Simpson R.M.C., Mayer K.J., Garofalo L.A., Moore A.N., Mora Garcia S.L., Or V.W., Srinivasan S., Farhan M. (2021). Acidity across the Interface from the Ocean Surface to Sea Spray Aerosol. Proc. Natl. Acad. Sci. USA.

[170] Sing K.S.W., Madeley J.D. (2007). The Surface Properties of Silica Gels. I. Importance of pH in the Preparation from Sodium Silicate and Sulphuric Acid. J. Appl. Chem..

[171] Balköse D. (2007). Effect of Preparation pH on Properties of Silica Gel. J. Chem. Technol. Biotechnol..

[172] Ülkü S., Balköse D., Baltacboğlu H. (1993). Effect of Preparation pH on Pore Structure of Silica Gels. Colloid Polym. Sci..

[173] Toyama Y., Sahara R., Iino Y., Kubota K. (2011). PH Dependence of Rheological Properties of Gelatin Gel Mixed with Agar or Agarose. Trans. Mater. Res. Soc. Jpn..

[174] Naser J., Mjalli F., Jibril B., Al-Hatmi S., Gano Z. (2013). Potassium Carbonate as a Salt for Deep Eutectic Solvents. Int. J. Chem. Eng. Appl..

[175] Skulcova A., Russ A., Jablonsky M., Sima J. (2018). The pH Behavior of Seventeen Deep Eutectic Solvents. BioResources.

[176] Toews K.L., Shroll R.M., Wai C.M., Smart N.G. (1995). PH-Defining Equilibrium between Water and Supercritical CO2. Influence on SFE of Organics and Metal Chelates. Anal. Chem..

[177] Goertz M.P., Goyal N., Montano G.A., Bunker B.C. (2011). Lipid Bilayer Reorganization under Extreme pH Conditions. Langmuir.

[178] Petelska A.D., Figaszewski Z.A. (2000). Effect of pH on the Interfacial Tension of Lipid Bilayer Membrane. Biophys. J..

[179] Ryan D.F., Kahler D.M. (1987). Geochemical and Mineralogical Indications of pH in Lakes and Soils in Central New Hampshire in the Early Holocene1. Limnol. Oceanogr..

[180] Hutchison W., Finch A.A., Borst A.M., Marks M.A.W., Upton B.G.J., Zerkle A.L., Stüeken E.E., Boyce A.J. (2021). Mantle Sources and Magma Evolution in Europe’s Largest Rare Earth Element Belt (Gardar Province, SW Greenland): New Insights from Sulfur Isotopes. Earth Planet. Sci. Lett..

[181] Sleep N.H., Bird D.K., Pope E.C. (2011). Serpentinite and the Dawn of Life. Philos. Trans. R. Soc. Lond. B Biol. Sci..

[182] Rohrback G.H., Cady G.H. (1951). The Liquid—Vapor Equilibrium of the System Tungsten Hexafluoride-Perfluorocycyclopentane1. J. Am. Chem. Soc..

[183] Gorgolis G., Galiotis C. (2017). Graphene Aerogels: A Review. 2D Mater..

[184] Mallamace F., Branca C., Broccio M., Corsaro C., Mou C.-Y., Chen S.-H. (2007). The Anomalous Behavior of the Density of Water in the Range 30 K < T < 373K. Proc. Natl. Acad. Sci. USA.

[185] Schön J.H. (2015). Density. Developments in Petroleum Science.

[186] Minic Z., Thongbam P.D. (2011). The Biological Deep Sea Hydrothermal Vent as a Model to Study Carbon Dioxide Capturing Enzymes. Mar. Drugs.

[187] Tanaka M., Girard G., Davis R., Peuto A., Bignell N. (2001). Recommended Table for the Density of Water between 0 C and 40 C Based on Recent Experimental Reports. Metrologia.

[188] Sarangi B., Aggarwal S.G., Sinha D., Gupta P.K. (2016). Aerosol Effective Density Measurement Using Scanning Mobility Particle Sizer and Quartz Crystal Microbalance with the Estimation of Involved Uncertainty. Atmos. Meas. Tech..

[189] Sofieva S., Asmi E., Atanasova N.S., Heikkinen A.E., Vidal E., Duplissy J., Romantschuk M., Kouznetsov R., Kukkonen J., Bamford D.H. (2022). Effects of Temperature and Salinity on Sea-Spray-Aerosol Formation Simulated with a Bubble-Generating Chamber. Atmos. Meas. Tech. Discuss..

[190] Timco G.W., Frederking R.M.W. (1996). A Review of Sea Ice Density. Cold Reg. Sci. Technol..

[191] Hou X.-J., Yu L.-Y., Wang Y.-X., Wu K.-J., He C.-H. (2021). Comprehensive Prediction of Densities for Deep Eutectic Solvents: A New Bonding-Group Interaction Contribution Scheme. Ind. Eng. Chem. Res..

[192] Yam H., Schmitt D.R. CO Rock Physics: A Laboratory Study. Proceedings of the Recovery–Joint CSPG CSEG CWLS Annual Convention.

[193] Guo Z., Rüpke L., Tao C. (2020). *HydrothermalFoam* v1.0: A 3-D Hydrothermal Transport Model for Natural Submarine Hydrothermal Systems. Geosci. Model Dev..

[194] Lewis R.A. (2016). Hawley’s Condensed Chemical Dictionary.

[195] Iravani M.A., Deparis J., Davarzani H., Colombano S., Guérin R., Maineult A. (2020). The Influence of Temperature on the Dielectric Permittivity and Complex Electrical Resistivity of Porous Media Saturated with DNAPLs: A Laboratory Study. J. Appl. Geophys..

[196] Rajput M.K., Konwar M., Sarma D. (2021). Hydrophobic Natural Deep Eutectic Solvent THY-DA as Sole Extracting Agent for Arsenic (III) Removal from Aqueous Solutions. Environ. Technol. Innov..

[197] Kim S., Huang J., Lee Y., Dutta S., Yoo H.Y., Jung Y.M., Jho Y., Zeng H., Hwang D.S. (2016). Complexation and Coacervation of like-Charged Polyelectrolytes Inspired by Mussels. Proc. Natl. Acad. Sci. USA.

[198] Earle S. (2019). Physical Geology.

[199] Zhuang B., Ramanauskaite G., Koa Z.Y., Wang Z.-G. (2021). Like Dissolves like: A First-Principles Theory for Predicting Liquid Miscibility and Mixture Dielectric Constant. Sci. Adv..

[200] Griffiths T.R., Pugh D.C. (1979). Correlations among Solvent Polarity Scales, Dielectric Constant and Dipole Moment, and a Means to Reliable Predictions of Polarity Scale Values from Cu. Coord. Chem. Rev..

[201] Ahmad Z. (2012). Polymer Dielectric Materials. Dielectric Material.

[202] Kato C., Nishihara S., Tsunashima R., Tatewaki Y., Okada S., Ren X.-M., Inoue K., Long D.-L., Cronin L. (2013). Quick and Selective Synthesis of Li6[α-P2W18O62]·28H2O Soluble in Various Organic Solvents. Dalton Trans..

[203] Mayer C., Schreiber U., Dávila M.J. (2017). Selection of Prebiotic Molecules in Amphiphilic Environments. Life.

[204] Klein L., Swift C. (1977). An Improved Model for the Dielectric Constant of Sea Water at Microwave Frequencies. IEEE J. Ocean. Eng..

[205] Malmberg C.G., Maryott A.A. (1956). Dielectric Constant of Water from 0 C to 100 C. J. Res. Natl. Bur. Stand..

[206] Davison S.W., Gentry J.W. (1985). Differences in Diffusion Charging of Dielectric and Conducting Ultrafine Aerosols. Aerosol Sci. Technol..

[207] Hrubesh L.W., Pekala R.W. (1994). Dielectric Properties and Electronic Applications of Aerogels. Sol-Gel Processing and Applications.

[208] Aragones J.L., MacDowell L.G., Vega C. (2011). Dielectric Constant of Ices and Water: A Lesson about Water Interactions. J. Phys. Chem. A.

[209] Essex J.W., Jorgensen W.L. (1995). Dielectric Constants of Formamide and Dimethylformamide via Computer Simulation. J. Phys. Chem..

[210] Bass S.J., Nathan W.I., Meighan R.M., Cole R.H. (1964). Dielectric Properties of Alkyl Amides. II. Liquid Dielectric Constant and Loss. J. Phys. Chem..

[211] Leeke G., Santos R., Al-Duri B., Seville J., Smith C., Holmes A.B. (2005). Solubilities of 4-Phenyltoluene, Phenylboric Acid, Biphenyl, and Iodobenzene in Carbon Dioxide from Measurements of the Relative Permittivity. J. Chem. Eng. Data.

[212] Li Z.X., Zhou J., Guo X.S., Ji B.B., Zhou W., Li D.H. (2018). Terahertz Spectral Properties of Coal Tar. J. Appl. Spectrosc..

[213] Gramse G., Dols-Perez A., Edwards M.A., Fumagalli L., Gomila G. (2013). Nanoscale Measurement of the Dielectric Constant of Supported Lipid Bilayers in Aqueous Solutions with Electrostatic Force Microscopy. Biophys. J..

[214] Dilger J.P., McLaughlin S.G., McIntosh T.J., Simon S.A. (1979). The Dielectric Constant of Phospholipid Bilayers and the Permeability of Membranes to Ions. Science.

[215] Yewdall N.A., André A.A.M., Lu T., Spruijt E. (2021). Coacervates as Models of Membraneless Organelles. Curr. Opin. Colloid Interface Sci..

[216] Takubo J., Ukai Y., Kuo C.C. (1953). On the dielectric constants of rocks. Mineral. J..

[217] Pan D., Spanu L., Harrison B., Sverjensky D.A., Galli G. (2013). Dielectric Properties of Water under Extreme Conditions and Transport of Carbonates in the Deep Earth. Proc. Natl. Acad. Sci. USA.

[218] Attinger D., Frankiewicz C., Betz A.R., Schutzius T.M., Ganguly R., Das A., Kim C.-J., Megaridis C.M. (2014). Surface Engineering for Phase Change Heat Transfer: A Review. MRS Energy Sustain..

[219] Petrov O., Furó I. (2011). A Study of Freezing–melting Hysteresis of Water in Different Porous Materials. Part I: Porous Silica Glasses. Microporous Mesoporous Mater..

[220] Budisa N., Schulze-Makuch D. (2014). Supercritical Carbon Dioxide and Its Potential as a Life-Sustaining Solvent in a Planetary Environment. Life.

[221] Schreiber U., Locker-Grütjen O., Mayer C. (2012). Hypothesis: Origin of Life in Deep-Reaching Tectonic Faults. Orig. Life Evol. Biosph..

[222] Zhang S.J., Duzdevich D., Ding D., Szostak J.W. (2022). Freeze-Thaw Cycles Enable a Prebiotically Plausible and Continuous Pathway from Nucleotide Activation to Nonenzymatic RNA Copying. Proc. Natl. Acad. Sci. USA.

[223] Mutschler H., Wochner A., Holliger P. (2015). Freeze-Thaw Cycles as Drivers of Complex Ribozyme Assembly. Nat. Chem..

[224] Jia T.Z., Fraccia T.P. (2020). Liquid Crystal Peptide/DNA Coacervates in the Context of Prebiotic Molecular Evolution. Crystals.

[225] Lu T., Nakashima K.K., Spruijt E. (2021). Temperature-Responsive Peptide-Nucleotide Coacervates. J. Phys. Chem. B.

[226] Chang H. (2008). The Myth of the Boiling Point. Sci. Prog..

[227] Ming F., Chen L., Li D., Du C. (2020). Investigation into Freezing Point Depression in Soil Caused by NaCl Solution. Water.

[228] Miyake Y. (1939). Chemical Studies of the Western Pacific Ocean. III. Freezing Point, Osmotic Pressure, Boiling Point, and Vapour Pressure of Sea Water. Bull. Chem. Soc. Jpn..

[229] Rosen J.M. (1971). The Boiling Point of Stratospheric Aerosols. J. Appl. Meteorol..

[230] DeMott P.J., Hill T.C.J., McCluskey C.S., Prather K.A., Collins D.B., Sullivan R.C., Ruppel M.J., Mason R.H., Irish V.E., Lee T. (2016). Sea Spray Aerosol as a Unique Source of Ice Nucleating Particles. Proc. Natl. Acad. Sci. USA.

[231] Wypych G. (2016). Handbook of Fillers.

[232] Saladino R., Crestini C., Ciciriello F., Pino S., Costanzo G., Di Mauro E. (2009). From Formamide to RNA: The Roles of Formamide and Water in the Evolution of Chemical Information. Res. Microbiol..

[233] Michael T. (2013). Formamide [MAK Value Documentation, 2013]. The MAK-Collection for Occupational Health and Safety.

[234] Clayton G.D. (1981). Patt’s Industrial Hygiene and Toxicology: Toxicology.

[235] Chapman J.B., Runyon S.E., Shields J.E., Lawler B.L., Pridmore C.J., Scoggin S.H., Swaim N.T., Trzinski A.E., Wiley H.N., Barth A.P. (2021). The North American Cordilleran Anatectic Belt. Earth Sci. Rev..

[236] Kennedy G.C., Higgins G.H. (1972). Melting Temperatures in the Earth’s Mantle. Developments in Geotectonics.

[237] Canıaz R.O., Erkey C. (2014). Process Intensification for Heavy Oil Upgrading Using Supercritical Water. Chem. Eng. Res. Des..

[238] Marinos-Kouris D., Krokida M., Oreopoulou V. (2006). Frying of Foods. Handbook of Industrial Drying.

[239] Martinotti C., Ruiz-Perez L., Deplazes E., Mancera R.L. (2020). Molecular Dynamics Simulation of Small Molecules Interacting with Biological Membranes. Chemphyschem.

[240] Kalepu S., Sunilkumar K.T., Betha S., Mohanvarma M. (2013). Liposomal Drug Delivery System—A Comprehensive Review. Int. J. Drug Dev. Res..

[241] Lowry C.A., Kay L.M. (2007). Chemical Factors Determine Olfactory System Beta Oscillations in Waking Rats. J. Neurophysiol..

[242] Chamberlin J.C. (1925). Heavy Mineral Oil as a Permanent Non-Volatile Preservative for Valuable Biological Material. Science.

[243] Gutmann F., Simmons L.M. (1950). A Theoretical Basis for the Antoine Vapor Pressure Equation. J. Chem. Phys..

[244] Meyers C.H. (2018). The Vapor Pressure of Liquid and Solid Carbon Dioxide (Classic Reprint).

[245] Orthoefer F.T., List G.R. (2007). Dynamics of Frying. Deep Frying.

[246] Mishra V.K., Temelli F., Ooraikul B. (1994). Vapor Pressure of Fatty Acid Esters: Correlation and Estimation. J. Food Eng..

[247] Bonnell D.G.R. (1932). Studies in Gels III. Vapour Pressure of Silica Gels. Trans. Faraday Soc..

[248] Wexler A. (1977). Vapor Pressure Formulation for Ice. J. Res. Natl. Bur. Stand. A Phys. Chem..

[249] Girnik I., Aristov Y. (2020). An Aqueous CaCl_2_ Solution in the Condenser/evaporator instead of Pure Water: Application for the New Adsorptive Cycle “heat from Cold”. Energies.

[250] Xin K., Roghair I., Gallucci F., van Sint Annaland M. (2021). Total Vapor Pressure of Hydrophobic Deep Eutectic Solvents: Experiments and Modelling. J. Mol. Liq..

[251] Bockish M. (1998). Composition, Structure, Physical Data, and Chemical Reactions of Fats and Oils, Their Derivatives, and Their Associates. Fats and Oils Handbook.

[252] Matricarde Falleiro R.M., Akisawa Silva L.Y., Meirelles A.J.A., Krähenbühl M.A. (2012). Vapor Pressure Data for Fatty Acids Obtained Using an Adaptation of the DSC Technique. Thermochim. Acta.

[253] Van Lente J., Pazos Urrea M., Brouwer T., Schuur B., Lindhoud S. (2021). Complex Coacervates as Extraction Media. Green Chem..

[254] Fegley B., Schaefer L., Kargel J.S. (2003). Vapor Pressure, Vapor Composition and Fractional Vaporization of High Temperature Lavas on Io. LPI Contrib..

[255] Guo Z., Qin X., Zhang Y., Niu C., Wang D., Ling Y. (2021). Numerical Investigation of the Effect of Heterogeneous Pore Structures on Elastic Properties of Tight Gas Sandstones. Front. Earth Sci..

[256] Molina O., Vilarrasa V., Zeidouni M. (2017). Geologic Carbon Storage for Shale Gas Recovery. Energy Procedia.

[257] Lowe D.R., Byerly G.R. (2018). The Terrestrial Record of Late Heavy Bombardment. New Astron. Rev..

[258] Lamour S., Pallmann S., Haas M., Trapp O. (2019). Prebiotic Sugar Formation Under Nonaqueous Conditions and Mechanochemical Acceleration. Life.

[259] Hansma H.G. (2022). Potassium at the Origins of Life: Did Biology Emerge from Biotite in Micaceous Clay?. Life.

[260] Shashi Menon E. (2011). Pipeline Planning and Construction Field Manual.

[261] Lei J., Liu Z., Yeo J., Ng T.Y. (2013). Determination of the Young’s Modulus of Silica Aerogels—An Analytical–numerical Approach. Soft Matter.

[262] Phair J.W., Tkachev S.N., Manghnani M.H., Livingston R.A. (2005). Elastic and Structural Properties of Alkaline-Calcium Silica Hydrogels. J. Mater. Res..

[263] Neumeier J.J. (2018). Elastic Constants, Bulk Modulus, and Compressibility of H_2_O Ice Ihfor the Temperature Range 50 K–273 K. J. Phys. Chem. Ref. Data.

[264] Schulson E.M. (1999). The Structure and Mechanical Behavior of Ice. JOM.

[265] Arriaga M.-C.S. Supercritical Thermodynamics of the Rock/Fluid Geothermal System. Proceedings of the World Geothermal Congress 2020+1.

[266] Lumley D., Sherlock D., Daley T., Huang L., Lawton D., Masters R., Verliac M., White D. (2010). Highlights of the 2009 SEG Summer Research Workshop on CO_2_ Sequestration. Lead. Edge.

[267] Terzi M.M., Deserno M., Nagle J.F. (2019). Mechanical Properties of Lipid Bilayers: A Note on the Poisson Ratio. Soft Matter.

[268] Picas L., Rico F., Scheuring S. (2012). Direct Measurement of the Mechanical Properties of Lipid Phases in Supported Bilayers. Biophys. J..

[269] Momeni Bashusqeh S., Rastgoo A. (2016). Elastic Modulus of Free-Standing Lipid Bilayer. Soft Mater..

[270] Marsden L.H., Neuberg J.W., Thomas M.E., Mothes P.A., Ruiz M.C. (2019). Combining Magma Flow and Deformation Modeling to Explain Observed Changes in Tilt. Front. Earth Sci..

[271] Heap M.J., Faulkner D.R., Meredith P.G., Vinciguerra S. (2010). Elastic Moduli Evolution and Accompanying Stress Changes with Increasing Crack Damage: Implications for Stress Changes around Fault Zones and Volcanoes during Deformation. Geophys. J. Int..

[272] Gutenberg B. (1959). Elastic Constants, and Elastic Processes in the Earth. International Geophysics.

[273] Burtch N.C., Heinen J., Bennett T.D., Dubbeldam D., Allendorf M.D. (2018). Mechanical Properties in Metal–Organic Frameworks: Emerging Opportunities and Challenges for Device Functionality and Technological Applications. Adv. Mater..

[274] Gough D.O. (1981). Solar Interior Structure and Luminosity Variations. Physics of Solar Variations.

[275] Ranjan S., Sasselov D.D. (2016). Influence of the UV Environment on the Synthesis of Prebiotic Molecules. Astrobiology.

[276] Ritson D., Sutherland J.D. (2012). Prebiotic Synthesis of Simple Sugars by Photoredox Systems Chemistry. Nat. Chem..

[277] Zang X., Ueno Y., Kitadai N. (2022). Photochemical Synthesis of Ammonia and Amino Acids from Nitrous Oxide. Astrobiology.

[278] Rastogi R.P., Richa, Kumar A., Tyagi M.B., Sinha R.P. (2010). Molecular Mechanisms of Ultraviolet Radiation-Induced DNA Damage and Repair. J. Nucleic Acids.

[279] Santos A.L., Moreirinha C., Lopes D., Esteves A.C., Henriques I., Almeida A., Domingues M.R.M., Delgadillo I., Correia A., Cunha A. (2013). Effects of UV Radiation on the Lipids and Proteins of Bacteria Studied by Mid-Infrared Spectroscopy. Environ. Sci. Technol..

[280] Indriolo N., McCall B.J. (2013). Cosmic-Ray Astrochemistry. Chem. Soc. Rev..

[281] Bertaina M., Apel W.D., Arteaga-Velázquez J.C., Bekk K., Blümer J., Bozdog H., Brancus I.M., Buchholz P., Cantoni E., Chiavassa A. (2011). The Cosmic Ray Energy Spectrum in the Range 10^16^–10^18^ eV Measured by KASCADE-Grande. Astrophys. Space Sci. Trans..

[282] Airapetian V.S., Glocer A., Gronoff G., Hébrard E., Danchi W. (2016). Prebiotic Chemistry and Atmospheric Warming of Early Earth by an Active Young Sun. Nat. Geosci..

[283] Ebisuzaki T., Maruyama S. (2017). Nuclear Geyser Model of the Origin of Life: Driving Force to Promote the Synthesis of Building Blocks of Life. Geosci. Front..

[284] Adam Z.R., Hongo Y., Cleaves H.J., Yi R., Fahrenbach A.C., Yoda I., Aono M. (2018). Estimating the Capacity for Production of Formamide by Radioactive Minerals on the Prebiotic Earth. Sci. Rep..

[285] Coogan L. (2009). Did Natural Reactors Form as a Consequence of the Emergence of Oxygenic Photosynthesis during the Archean?. GSA Today.

